# One-size-fits-all versus risk-category-based screening interval strategies for cardiovascular disease prevention in Chinese adults: a prospective cohort study

**DOI:** 10.1016/j.lanwpc.2024.101140

**Published:** 2024-07-16

**Authors:** Zhijia Sun, Yu Ma, Canqing Yu, Dianjianyi Sun, Yuanjie Pang, Pei Pei, Ling Yang, Yiping Chen, Huaidong Du, Hao Zhang, Xiaoming Yang, Maxim Barnard, Robert Clarke, Junshi Chen, Zhengming Chen, Liming Li, Jun Lv, China Kadoorie Biobank Collaborative Group, Junshi Chen, Zhengming Chen, Robert Clarke, Rory Collins, Liming Li, Jun Lv, Richard Peto, Robin Walters, Daniel Avery, Maxim Barnard, Derrick Bennett, Lazaros Belbasis, Ruth Boxall, Ka Hung Chan, Yiping Chen, Zhengming Chen, Charlotte Clarke, Johnathan Clarke, Robert Clarke, Huaidong Du, Ahmed Edris Mohamed, Hannah Fry, Simon Gilbert, Pek Kei Im, Andri Iona, Maria Kakkoura, Christiana Kartsonaki, Hubert Lam, Kuang Lin, James Liu, Mohsen Mazidi, Iona Millwood, Sam Morris, Qunhua Nie, Alfred Pozarickij, Maryanm Rahmati, Paul Ryder, Saredo Said, Dan Schmidt, Becky Stevens, Iain Turnbull, Robin Walters, Baihan Wang, Lin Wang, Neil Wright, Ling Yang, Xiaoming Yang, Pang Yao, Xiao Han, Can Hou, Qingmei Xia, Chao Liu, Jun Lv, Pei Pei, Dianjianyi Sun, Canqing Yu, Lang Pan, Zengchang Pang, Ruqin Gao, Shanpeng Li, Haiping Duan, Shaojie Wang, Yongmei Liu, Ranran Du, Yajing Zang, Liang Cheng, Xiaocao Tian, Hua Zhang, Yaoming Zhai, Feng Ning, Xiaohui Sun, Feifei Li, Silu Lv, Junzheng Wang, Wei Hou, Wei Sun, Shichun Yan, Xiaoming Cui, Chi Wang, Zhenyuan Wu, Yanjie Li, Quan Kang, Huiming Luo, Tingting Ou, Xiangyang Zheng, Zhendong Guo, Shukuan Wu, Yilei Li, Huimei Li, Ming Wu, Yonglin Zhou, Jinyi Zhou, Ran Tao, Jie Yang, Jian Su. Fang Liu, Jun Zhang, Yihe Hu, Yan Lu, Liangcai Ma, Aiyu Tang, Shuo Zhang, Jianrong Jin, Jingchao Liu, Mei Lin, Zhenzhen Lu, Lifang Zhou, Changping Xie, Jian Lan, Tingping Zhu, Yun Liu, Liuping Wei, Liyuan Zhou, Ningyu Chen, Yulu Qin, Sisi Wang, Xianping Wu, Ningmei Zhang, Xiaofang Chen, Xiaoyu Chang, Mingqiang Yuan, Xia Wu, Xiaofang Chen, Wei Jiang, Jiaqiu Liu, Qiang Sun, Faqing Chen, Xiaolan Ren, Caixia Dong, Hui Zhang, Enke Mao, Xiaoping Wang, Tao Wang, Xi Zhang, Kai Kang, Shixian Feng, Huizi Tian, Lei Fan, XiaoLin Li, Huarong Sun, Pan He, Xukui Zhang, Min Yu, Ruying Hu, Hao Wang, Xiaoyi Zhang, Yuan Cao, Kaixu Xie, Lingli Chen, Dun Shen, Xiaojun Li, Donghui Jin, Li Yin, Huilin Liu, Zhongxi Fu, Xin Xu, Hao Zhang, Jianwei Chen, Yuan Peng, Libo Zhang, Chan Qu

**Affiliations:** a Department of Epidemiology & Biostatistics, School of Public Health, Peking University, Beijing, 100191, China; b Peking University Center for Public Health and Epidemic Preparedness & Response, Beijing, 100191, China; c Key Laboratory of Epidemiology of Major Diseases (Peking University), Ministry of Education, Beijing, China; d Clinical Trial Service Unit & Epidemiological Studies Unit (CTSU), Nuffield Department of Population Health, University of Oxford, United Kingdom; e Liuyang CDC, Hunan, 410300, China; f China National Center for Food Safety Risk Assessment, Beijing, China; g State Key Laboratory of Vascular Homeostasis and Remodeling, Peking University, Beijing, China

**Keywords:** Cardiovascular disease, Screening, Primary prevention

## Abstract

**Background:**

In non-high-risk individuals, risk-category-based atherosclerotic cardiovascular disease (ASCVD) screening strategies may be more cost-effective than one-size-fits-all approaches. However, current decisions are constrained by a lack of research evidence. We aimed to explore appropriate risk-category-based screening interval strategies for non-high-risk individuals in ASCVD primary prevention in the Chinese population.

**Methods:**

We used data from 28,624 participants in the China Kadoorie Biobank (CKB) who had completed at least two field surveys. The risk assessment tools were the 10-year ASCVD risk prediction models developed based on the CKB cohort. We constructed multistate Markov models to model disease progression and estimate transition probabilities between different risk categories. The total person-years spent unidentified in the high-risk state over a 10-year period were calculated for each screening interval protocol. We also estimated the number of ASCVD events prevented, quality-adjusted life years (QALYs) gained, and costs saved when compared to the 3-yearly screening protocol.

**Findings:**

When compared to the uniform 3-yearly protocol, most risk-category-based screening interval protocols would identify more high-risk individuals timely, thus preventing more ASCVD events and gaining QALYs. A few of them would reduce total health-care costs. The protocol, which used 6-year, 3-year, and 2-year screening intervals for low-risk, intermediate-low-risk, and intermediate-high risk individuals, was optimal, and would reduce the person-years spent unidentified in the high-risk category by 17.9% (95% CI: 13.1%–21.9%), thus preventing an estimated 113 thousand (95% CI: 83–138) hard ASCVD events for Chinese adults aged 30–79 over a 10-year period. When using a lower cost of statin therapy, more screening protocols would gain QALYs while saving costs.

**Interpretation:**

For the primary prevention of ASCVD, risk-category-based screening protocols outperformed the one-size-fits-all approach in the Chinese population.

**Funding:**

This work was supported by 10.13039/501100001809National Natural Science Foundation of China (82192904, 82388102, 82192900) and grants (2023YFC2509400) from the National Key R&D Program of China. The CKB baseline survey and the first re-survey were supported by a grant from the 10.13039/501100017647Kadoorie Charitable Foundation in Hong Kong. The long-term follow-up is supported by grants from the UK 10.13039/100010269Wellcome Trust (212946/Z/18/Z, 202922/Z/16/Z, 104085/Z/14/Z, 088158/Z/09/Z), grants (2016YFC0900500) from the National Key R&D Program of China, 10.13039/501100001809National Natural Science Foundation of China (81390540, 91846303, 81941018), and Chinese Ministry of Science and Technology (2011BAI09B01).

**Research in context**

**Evidence before this study**

Current guidelines recommend regular risk assessment for individuals without cardiovascular disease for primary prevention, but screening intervals are mostly based on expert recommendations rather than evidence. We identified published studies using the terms “cardiovascular disease” AND “screening” AND “prevention” AND “risk assessment” in PubMed and Embase from inception to May 2024. Only one UK study explored the optimal screening interval for non-high-risk individuals. However, the benefits and costs of atherosclerotic cardiovascular disease (ASCVD) risk screening interval protocols may vary depending on population and health resources. There is no evidence for the use of risk-category-based screening interval strategies in resource-limited populations.

**Added value of this study**

The current study in the Chinese population found that, when compared to the one-size-fits-all 3-yearly screening protocol, most risk-category-based screening interval protocols would identify more high-risk individuals timely, thus preventing more ASCVD events and gaining quality-adjusted life years. A few of them would reduce total health-care costs. The protocol, which used 6-year, 3-year, and 2-year screening intervals for low-risk, intermediate-low-risk, and intermediate-high-risk individuals, was optimal for Chinese adults aged 30–79 over a 10-year period.

**Implications of all the available evidence**

Evidence from this study, as well as the previous UK study, supports that risk-category-based screening interval strategies are more cost-effective than a one-size-fits-all approach in both resource-rich and resource-limited populations.

## Introduction

In the primary prevention of cardiovascular disease, absolute risk assessment is critical for identifying high-risk individuals and initiating lifestyle interventions and statin therapy.^1^, ^2^, ^3^, ^4^, ^5^, ^6^ Non-high-risk groups must conduct regular risk assessments in order to identify new high-risk groups in a timely manner. However, determining the optimal screening interval faces a major challenge.^7^ Overuse of screening may have emotional, physical, and financial consequences for individuals, as well as strain the healthcare system. A too-long screening interval, on the other hand, may lead to missed opportunities for early intervention.^8^^,^^9^ There is currently limited research evidence to guide decisions on screening intervals.^10^ Clinical guidelines are primarily based on experience and expert consensus.^11^ Current guidelines differ in their recommendations for screening intervals for non-high-risk groups, such as every 4–6 years for individuals aged 20 and above in the United States,^2^ every 5 years for men over 40 and women over 50 in Europe,^3^ and every 2–3 years for individuals over 35 without cardiovascular risk factors in China.^4^

A previous study using data from two cohorts of Japanese and American adults found that individuals with a baseline risk of less than 5% had a 6.8% chance of crossing the 20% risk threshold during a 19-year follow-up and individuals with a baseline risk between 5.0% and 9.9% had a 9.1% chance of crossing the 20% risk threshold during an 8-year follow-up, implying that the screening interval can be extended for low-risk individuals.^12^ So far, only one UK study has explored the optimal screening interval based on risk categories of atherosclerotic cardiovascular disease (ASCVD).^13^ This study found that, compared with the 5-yearly screening strategy, adopting 7-year, 4-year, and 1-year screening intervals for low-risk, intermediate-low-risk, and intermediate-high-risk individuals, respectively, would perform better in terms of preventing ASCVD events and improving cost-effectiveness. However, the benefits and costs of ASCVD risk screening interval protocols may vary depending on population and health resources. Cardiovascular risk may be different, such as a higher incidence of stroke in China than in the Western population,^14^ and it can also progress at different rates in different populations. As a result, more research is needed to determine the appropriate interval for repeat ASCVD risk assessment, particularly in resource-limited populations.^13^

The purpose of this study was to compare the cost-effectiveness of the current recommendation of a uniform 3-yearly screening protocol versus risk-category-based screening interval strategies for non-high-risk individuals in cardiovascular disease prevention in the Chinese population.

## Methods

### Study design and population

The China Kadoorie Biobank (CKB) cohort consists of 512,723 participants recruited from 5 urban and 5 rural areas in China between 2004 and 2008. All participants were followed up through linkages with death and disease registries, health insurance records, and active follow-up to identify mortality, morbidity, and hospitalization events. The tenth revision of the International Classification of Diseases was used to code all documented events. The outcomes of interest in this study included all fatal and non-fatal myocardial infarction (MI, I21–I23), ischemic heart disease (IHD, I20–I25), and ischemic stroke (IS, I63), and death due to non-ASCVD causes up to December 31, 2018. The loss to follow-up at this point in time was 4027 (<1%). Since 2014, participant-matched medical records have been retrieved and adjudicated by an independent team of qualified cardiovascular specialists, blinded to participants' baseline information. By October 2018, of retrieved medical records of 33,515 IHD cases and 34,758 IS cases, the diagnosis was confirmed in 87.9% and 91.5%, respectively.^15^

Resurveys after baseline were conducted every 4–5 years. The rural village or urban residential committee was the primary sampling unit, and approximately 5% of participants were randomly selected by cluster sampling. The first resurvey conducted in 2008 had approximately 20,000 participants, and the second resurvey in 2013–2014 had approximately 25,000 participants. Participants completed an interviewer-administered laptop-based questionnaire, physical measurements, and blood sample collection by trained staff at each survey. All information was entered into a computer program with built-in functions to avoid missing items and minimize logic errors during the interview. Details of the study design and survey methods have been reported previously.^16^ CKB received ethical approvals from the Ethical Review Committee of the Chinese Center for Disease Control and Prevention (approval number 005/2004, 9th July 2004) and the Oxford Tropical Research Ethics Committee at the University of Oxford (approval number 025-04, 3rd February 2005). All participants provided written informed consent.

**Fig. 1:**
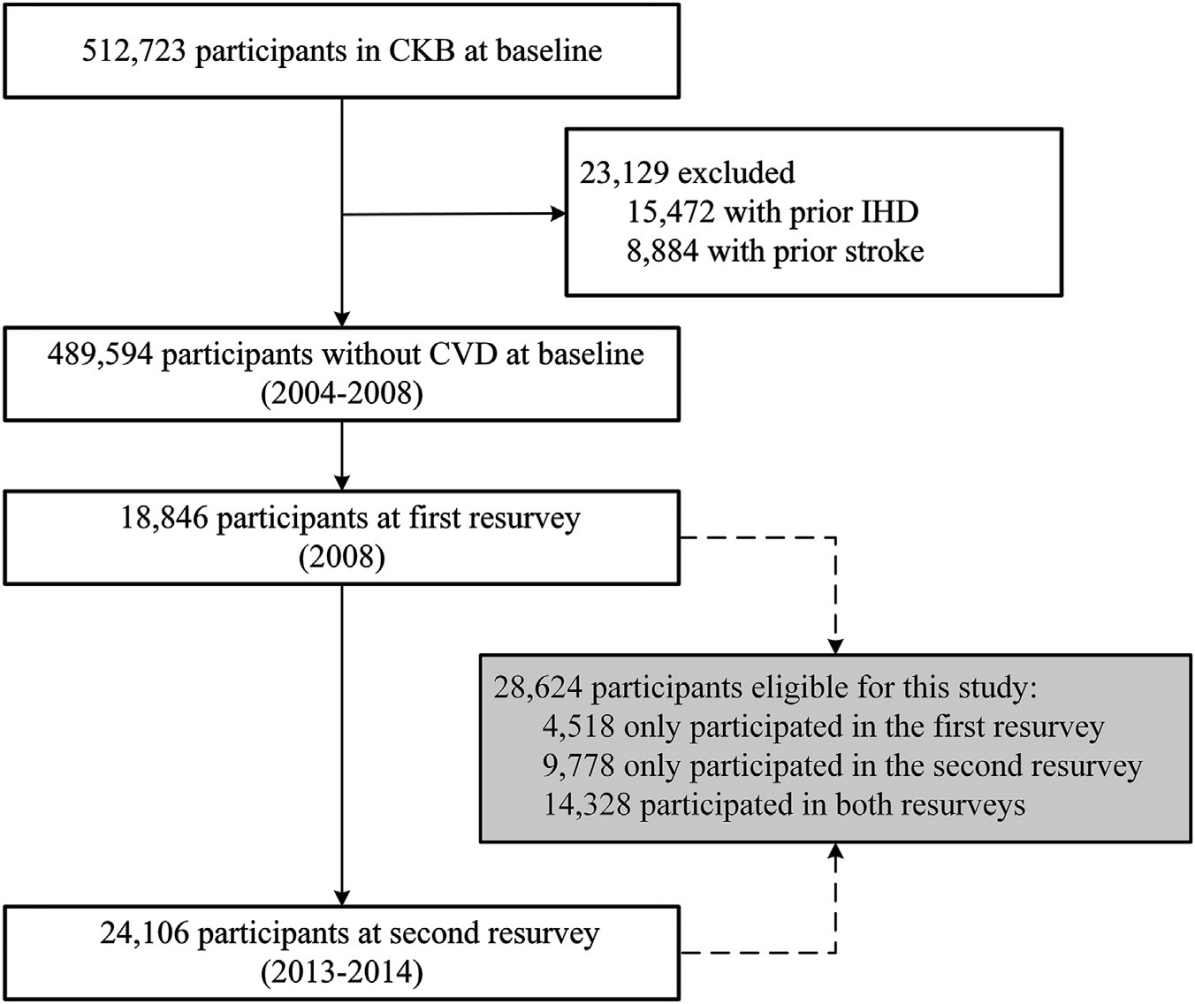
**Flow chart of participants in the study**. CKB, China Kadoorie Biobank; IHD, ischemic heart disease; CVD, cardiovascular disease.

We excluded participants with a history of heart disease (n = 15,472) or stroke (n = 8884) at baseline. Of the remaining 489,594 participants, 28,624 who had completed at least one resurvey were included in this study (Fig. 1).

### ASCVD risk assessment tools

In this study, we used 10-year ASCVD risk prediction models developed based on the CKB cohort as the risk assessment tools (CKB-ASCVD model). The sex-specific CKB-ASCVD model was developed with the following variables: age (years), systolic and diastolic blood pressure (mmHg), use of blood pressure-lowering medication (yes/no), current daily smoking (yes/no), self-reported history of diabetes (yes/no), and waist circumference (cm). The predictor definitions and model construction methods have been described in detail elsewhere.^15^ The details on the model development are shown in supplementary methods and eTables 1 and 2. Following the recalibration approach proposed by the WHO CVD Risk Chart Working Group,^17^ we further recalibrated the models based on the risk factor levels and observed 10-year ASCVD risks in each study area.

We defined two ASCVD outcomes: (1) soft ASCVD outcomes, including all fatal or non-fatal IHD and IS; and (2) hard ASCVD outcomes, including non-fatal MI, fatal IHD, and fatal or non-fatal IS.^18^ Prediction models developed for both outcomes performed well in terms of discrimination and calibration. The Harrell C for the soft outcome model was 0.776 (95% CI: 0.774–0.777) for women and 0.773 (0.770–0.775) for men. The Harrell C for the hard outcome model was 0.805 (95% CI: 0.802–0.807) for women and 0.795 (95% CI: 0.792–0.798) for men. The calibration performance of hard and soft outcome models is shown in eFigure 1. In this study, we used the hard outcome model as the primary analysis and soft outcome model as the secondary analysis.

### Statistical analysis

#### ASCVD risk classification

First, we used the CKB-ASCVD hard outcome model to predict 10-year risk of ASCVD at baseline and each resurvey among participants free of cardiovascular disease. The estimated ASCVD risk was categorized into three levels following the current Chinese guideline recommendation: low (<5.0%), intermediate (5.0–9.9%), and high risk (≥10.0%).^4^ We also classified the risk into four levels by using different combinations of cut-off values, including 2.5% and 5.0%, 2.5% and 7.5%, and 5.0% and 7.5%. For example, we used cut-offs of 2.5% and 5.0% to define low (<2.5%), intermediate-low (2.5%–4.9%), intermediate-high (5.0%–9.9%), and high risk (≥10.0%).

In the analyses of soft outcome model, we defined three risk categories as low (<10.0%), intermediate (10.0–19.9%), and high risk (≥20.0%). Three risk cut-off combinations, including 5.0% and 10.0%, 5.0% and 15.0%, and 10.0% and 15.0%, were used to create four risk categories.

#### Construction of the multistate model

**Fig. 2:**
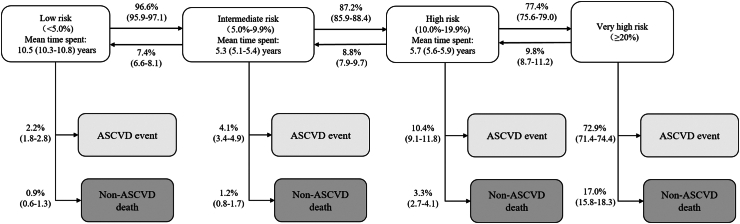
**Estimated mean sojourn time in each ASCVD 10-year-risk category and transition probabilities to the next risk category**. ASCVD, atherosclerotic cardiovascular disease. Data are estimates with 95% CIs. The 10-year risks were estimated by CKB-ASCVD hard outcome model.

We constructed a multistate Markov model to model the disease progression from different risk states to ASCVD events or death from non-ASCVD events (Fig. 2). In this multistate model, bidirectional transitions between non-high-risk states were allowed, and ASCVD events (including all fatal and non-fatal IHD and IS events) and non-ASCVD death were absorbing states. We estimated the transition probabilities between these health states over time and the average time spent in non-absorbing states using the R package “msm”.^19^ We used the “BFGS” quasi-Newton optimization algorithm to facilitate convergence, with a convergence criterion of 1e-16. The overall fit of the model is good, with a slight underestimation of low-risk state prevalence and an overestimation of high-risk state prevalence with enrollment time (eFigure 2).

#### Estimation of person-years spent unidentified in high-risk state

We defined 27 risk-category-based screening interval protocols, including 9 protocols with three risk categories (low, intermediate, and high) and 18 protocols with four risk categories (low, intermediate-low, intermediate-high, and high). Specifically, the reference protocol was the uniform 3-yearly screening protocol for low- and intermediate-risk individuals recommended by the Chinese guideline.^4^ For three risk categories, we set screening intervals ranging from 3 to 6 years for low-risk individuals and 1–2 years for intermediate-risk individuals, resulting in 8 protocols. For four risk categories, we set screening intervals ranging from 6 to 8 years, 3–5 years, and 1–2 years for low-risk, intermediate-low-risk, and intermediate-high-risk individuals, respectively, resulting in 18 protocols (eTable 3).

Referring to a previous study,^13^ we calculated the total person-years spent unidentified in the high-risk state over a 10-year period for each screening interval protocol using the number of people in non-high-risk states initially and transition probabilities from non-high-risk to high-risk states. We did not consider transitions from high-risk to non-high-risk states, assuming that high-risk individuals would receive statin therapy in accordance with guidelines.

To estimate potential benefits of risk-category-based screening interval strategy in the Chinese population, age- and sex-specific 10-year ASCVD risk distributions in the CKB cohort were applied to the Chinese population in 2019 (eTable 4).^20^ The population free of ASCVD was estimated using the prevalence of IHD and IS from the Global Burden of Disease (GBD) study 2019 (eTable 5).^21^ The estimated populations in different risk categories were the initial number of non-high-risk Chinese population (Table 1).

**Table 1:** Parameters used in the study.

	Value
Number of Chinese population in different risk score groups, million	
Risks estimated by CKB-ASCVD hard outcome model	
<2.5%	273.89
2.5%–4.9%	153.51
5.0%–7.4%	90.16
7.5%–9.9%	61.46
≥10.0%	244.05
Risks estimated by CKB-ASCVD soft outcome model	
<5.0%	271.66
5.0%–9.9%	172.20
10.0%–14.9%	102.24
15.0%–19.9%	67.28
≥20.0%	209.66
Parameters on statin treatment for high-risk individuals per 100,000 person-year	
QALYs gained	225.17
ASCVD events prevented	251.86
Diabetes events caused	85.81
Myopathy events caused	34.37
Parameters on statin treatment for high-risk individuals per person-year	
Incremental cost, 2019 CN¥	212.34
Low limit of the incremental cost, 2019 CN¥	70.78
Cost per health check, 2019 CN¥	30.00

CKB, China Kadoorie Biobank; ASCVD, atherosclerotic cardiovascular disease; QALY, quality-adjusted life year; CN¥, Chinese Yuan.

Parameters on benefits from statin treatment are derived from a cost-effectiveness analysis based on the China Multi-provincial Cohort Study, a nationwide and representative study in China. Health-check cost is derived from the registration fee for a hospital visit in China.

#### Estimation of costs and QALYs

We identified the incremental cost and effectiveness of statin therapy versus no treatment for high-risk individuals by referring to a previous cost-effectiveness analysis based on the China Multi-provincial Cohort Study (Table 1).^22^ We then multiplied these parameters by the person-years spent unidentified in the high-risk state with each screening protocol to estimate the ASCVD events prevented, quality-adjusted life years (QALYs) gained, and costs related to statin therapy.

According to the aforementioned study,^22^ the statin cost accounted for only a quarter of total costs for individuals taking low-dose statins, with the rest coming from other treatment-related costs like risk assessment, medical service, and adverse events screening and treatment. Given that purchasing drugs in primary care settings and using risk assessment tools without blood lipid testing could reduce costs and thus the incremental costs of statin therapy, we used the low limit of incremental cost in addition to the average incremental cost in the analysis.

The costs of periodic risk assessments over a 10-year period were estimated by multiplying the number of screenings by the cost of a health check. The costs of statin therapy and periodic risk assessments were added up to calculate the total costs of each screening interval protocol and thus the difference from the 3-yearly screening protocol. When compared with the 3-yearly screening protocol, we valued alternative protocols that would reduce the number of person-years spent unidentified in the high-risk state, prevent more ASCVD events, and gain more QALYs while reducing total costs. We considered the protocol that would prevent most ASCVD events while not increasing costs as the optimal strategy. We further repeated the analyses by sex.

Analyses were performed using R (version 4.1.0) and Stata (version 17.0).

### Role of the funding source

The funders had no role in the study design, data collection, data analysis and interpretation, writing of the report, or the decision to submit the article for publication.

## Results

### Overview of the study population

The median age at baseline of the 28,624 participants in this study was 51.1 years (IQR: 16.1), and 39.0% were men (Table 2). The average 10-year ASCVD risk level increased over time and was higher in men than in women (Table 2 and eTable 6). The proportion of participants in the high-risk category, as predicted by the hard outcome model, increased from 29.8% at baseline to 50.5% at the second resurvey.

**Table 2:** Characteristics of the study population.

	All	Men	Women
**Baseline characteristics**			
No. of participants	28,624	11,158 (39.0)	17,466 (61.0)
Age, years	51.1 (42.6–58.7)	51.8 (43.0–59.9)	50.6 (42.4–57.9)
Systolic blood pressure, mm Hg	128.0 (116.5–142.5)	129.5 (119.5–142.5)	126.5 (114.5–142.5)
Diastolic blood pressure, mm Hg	77.0 (70.0–84.5)	78.0 (71.0–86.0)	76.0 (69.5–83.5)
Use of blood pressure-lowering treatment, %	2997 (10.5)	1035 (9.3)	1962 (11.2)
Current daily smoker, %	6661 (23.3)	6309 (56.5)	352 (2.0)
Self-reported diabetes, %	756 (2.6)	288 (2.6)	468 (2.7)
Waist circumference, cm	78.9 (72.5–85.7)	81.0 (74.2–88.0)	77.5 (71.6–84.1)
**10-year risk category at baseline**			
Risk scores, %	5.0 (2.1–11.8)	6.8 (3.1–14.6)	4.1 (1.7–10.0)
Risk score grouping			
<2.5%	8381 (29.3)	2180 (19.5)	6201 (35.5)
2.5%–4.9%	5877 (20.5)	2260 (20.3)	3617 (20.7)
5.0%–7.4%	3423 (12.0)	1521 (13.6)	1902 (10.9)
7.5%–9.9%	2427 (8.5)	1063 (9.5)	1364 (7.8)
≥10.0%	8516 (29.8)	4134 (37.0)	4382 (25.1)
**10-year risk category at first resurvey**			
No. of participants	18,846	7375 (39.1)	11,471 (60.9)
Age, years	53.8 (45.2–61.6)	54.5 (45.4–63.2)	53.4 (45.1–60.7)
No. of participants without ASCVD	18,531	7243 (39.1)	11,288 (60.9)
Risk scores, %^a^	6.1 (2.6–13.9)	7.8 (3.5–16.5)	5.1 (2.2–12.4)
Risk score grouping^a^			
<2.5%	4359 (23.5)	1127 (15.6)	3232 (28.6)
2.5%–4.9%	3797 (20.5)	1440 (19.9)	2357 (20.9)
5.0%–7.4%	2274 (12.3)	958 (13.2)	1316 (11.7)
7.5%–9.9%	1663 (9.0)	726 (10.0)	937 (8.3)
≥10.0%	6438 (34.7)	2992 (41.3)	3446 (30.5)
**10-year risk category at second resurvey**			
No. of participants	24,106	9205 (38.2)	14,901 (61.8)
Age, years	58.9 (50.5–66.2)	59.6 (51.0–67.3)	58.5 (50.3–65.5)
No. of participants without ASCVD	22,420	8501 (37.9)	13,919 (62.1)
Risk scores, %^a^	10.1 (4.7–20.3)	12.6 (6.3–23.7)	8.8 (4.0–17.9)
Risk score grouping^a^			
<2.5%	2637 (11.8)	588 (6.9)	2049 (14.7)
2.5%–4.9%	3326 (14.8)	969 (11.4)	2357 (16.9)
5.0%–7.4%	2885 (12.9)	1072 (12.6)	1813 (13.0)
7.5%–9.9%	2256 (10.1)	844 (9.9)	1412 (10.1)
≥10.0%	11,316 (50.5)	5028 (59.1)	6288 (45.2)

ASCVD, atherosclerotic cardiovascular disease.

Data are numbers (percentages) or median (25th–75th percentile range) unless otherwise specified. The 10-year risks were estimated by CKB-ASCVD hard model.

a Risk scores were calculated and categorized in participants without ASCVD.

During a median follow-up of 12.8 years (IQR: 1.2), 5063 participants had incident ASCVD events, and 1802 died from non-ASCVD causes. Among the documented ASCVD events, there were 195 non-fatal MI, 2390 non-fatal non-MI IHD, 308 fatal IHD, 2807 non-fatal IS, and 111 fatal IS. Of the 5063 participants, 3921 (77.4%) participants were estimated to be in the high-risk category at their most recent survey before the occurrence of ASCVD events. There were 6387 participants in non-high-risk categories at baseline who progressed to the high-risk category during the follow-up period.

### Description of transitions between health states

Using the CKB-ASCVD hard outcome model, the estimated mean sojourn time spent in the low-risk category was 10.5 years (95% CI: 10.3–10.8) and 5.3 years (95% CI: 5.1–5.4) in the intermediate-risk category (Fig. 2). For participants at low, intermediate, and high risk, the probabilities to the next category of ASCVD events were 2.2%, 4.1%, and 10.4%, respectively. The transition probabilities to the high-risk category from the intermediate-risk category were significantly higher than those from the low-risk category (eFigure 3). Apart from transitions to higher-risk categories, 7.4% of participants at intermediate risk reversed to low risk, and 8.8% of high-risk participants reversed to intermediate risk. The description of transitions for four risk categories is shown in eFigure 4.

### Comparisons of risk-category-specific screening interval protocols

**Fig. 3:**
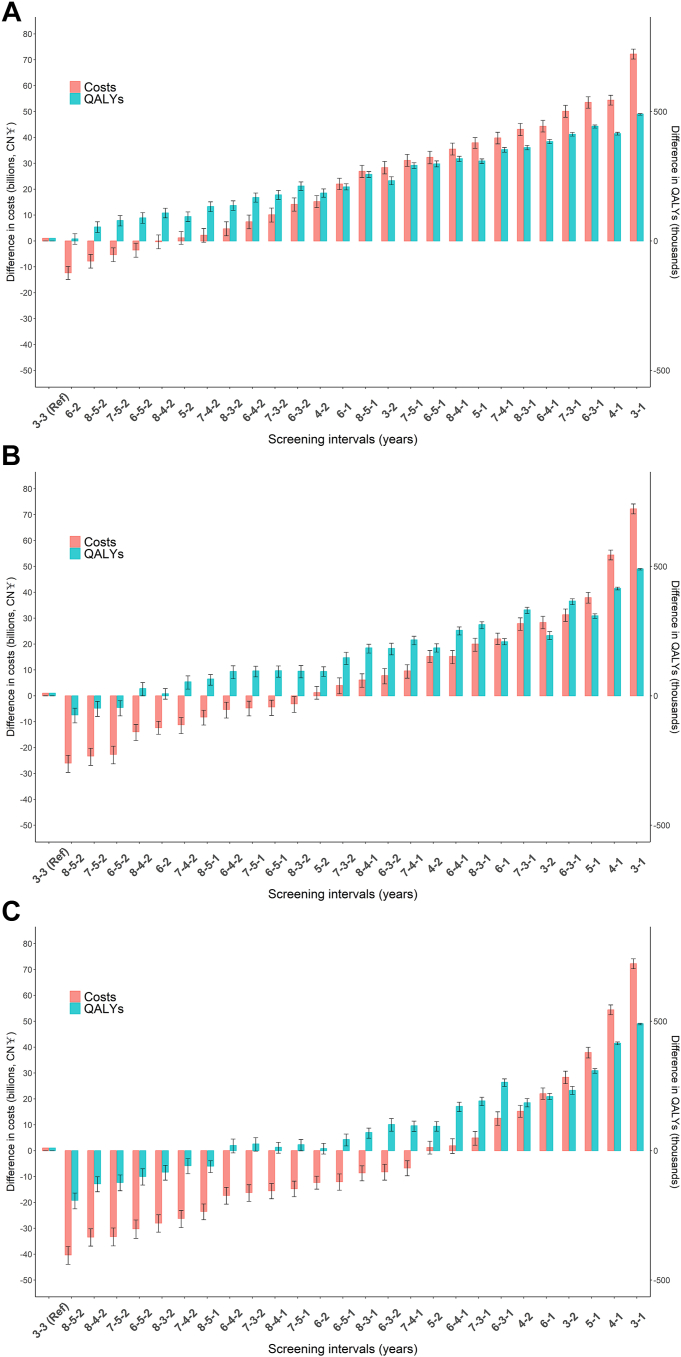
**Comparisons of all screening interval protocols with 3-yearly screening protocol with 10-year risk estimated by CKB-ASCVD hard outcome model**. QALY, quality-adjusted life year. Red bars represent differences in total health-care costs, and green bars represent differences in QALYs gained in the Chinese population over a 10-year period. Data are estimates with 95% CIs. In the three figures, three-risk categories were uniformly defined as low (<5.0%), intermediate (5.0%–9.9%), and high risk (≥10.0%). The risk cut-offs of the four-risk categories were different in the three figures, namely: (A) low (<2.5%), intermediate-low (2.5%–4.9%), intermediate-high (5.0%–9.9%), and high risk (≥10.0%); (B) low (<2.5%), intermediate-low (2.5%–7.4%), intermediate-high (7.5%–9.9%), and high risk (≥10.0%); (C) low (<5.0%), intermediate-low (5.0%–7.4%), intermediate-high (7.5%–9.9%), and high risk (≥10.0%).

Using the hard outcome model, most risk-category-based screening interval protocols would prevent more ASCVD events and gain QALYs by reducing person-years spent unidentified in the high-risk category when compared with the uniform 3-yearly screening interval protocol (eTable 7 and Fig. 3). However, only a few four-risk-category protocols outperformed the reference protocol in terms of QALY gains while reducing health-care costs. When using the soft outcome model, the 6–2 screening protocol (that is, 6-year and 2-year screening intervals for low-risk and intermediate-risk individuals, respectively) and a few four-risk-category protocols were better protocols than reference one (eTable 8 and eFigure 5).

When the cut-offs of 2.5% and 5.0% for four-risk-category classification were used, three screening protocols performed better: 8-5-2, 7-5-2, and 6-5-2 (eTable 7 and Fig. 3A). Similarly, when the cut-offs of 2.5% and 7.5% (Figs. 3B) and 5.0% and 7.5% (Fig. 3C) were used, there were 7 and 4 better protocols, respectively.

Among all alternative screening protocols that were better than the reference one, the protocol using 6-year, 3-year, and 2-year screening intervals for low-risk (<5.0%), intermediate-low-risk (5.0%–7.4%), and intermediate-high risk (7.5%–9.9%) individuals, respectively was optimal. Compared with the 3-yearly screening protocol, the 6-3-2 screening protocol for Chinese adults aged 30–79 would reduce the person-years spent unidentified in the high-risk category by 17.9% (95% CI: 13.1%–21.9%). Over a 10-year period, it would prevent an estimated 113 thousand (95% CI: 83–138) ASCVD events (1.95/100,000 person-years) with earlier statin intervention, cause 39 thousand (95% CI: 28–47) new diabetes and 15 thousand (95% CI: 11–19) myopathy events by statin treatment, gain 101 thousand (95% CI: 75–124) QALYs, and save total health-care costs by approximately 8.2 (95% CI: 5.3–11.4) billion Chinese Yuan. For the soft outcome model analysis, the 6-3-2 screening protocol with cut-offs of 10.0% and 15.0% was also optimal. The optimal strategy remained mostly unchanged in the sex-specific analyses; however, there were more screening strategies that outperformed the reference strategy in women than in men (eFigures 6 and 7).

When using the low limit of the incremental cost of statin therapy for high-risk individuals, more screening protocols would save costs (eTable 9 and eFigure 8). The 6-3-1 screening protocol with cut-offs of 5.0% and 7.5% was optimal.

## Discussion

In this study, we found that compared with the 3-yearly screening protocol for all non-high-risk individuals recommended by current Chinese guideline, risk-category-based screening protocols would reduce time spent unidentified in the high-risk category at a lower cost, thus preventing more ASCVD events and increasing QALYs through more timely preventive interventions. Among all 27 screening protocols, when the hard outcome model was used, the 6-year, 3-year, and 2-year screening intervals for low-risk, intermediate-low-risk, and intermediate-high-risk individuals, respectively, performed best with cut-offs of 5.0% and 7.5%. When the soft outcome model was used, the 6-year, 3-year, and 2-year screening interval protocol also performed best with cut-offs of 10% and 15%.

Previous studies have examined screening intervals for a single CVD risk factor by estimating short-term fluctuations and long-term changes. The findings indicated that for individuals with controlled blood pressure or lipid, the re-screening interval should be extended beyond three years.^23^, ^24^, ^25^, ^26^ Only one study explored the optimal screening intervals for different levels of overall ASCVD risk.^13^ Using data from the British Whitehall II study, this study involved 6964 participants aged 40–64 who had participated in up to five repeated screenings between 1991 and 2015 and used an ASCVD risk calculator that included blood lipid measurements. All 21 risk-category-based screening interval protocols outperformed the 5-yearly screening interval in terms of ASCVD events avoided and QALYs gained. Furthermore, 16 protocols were associated with costs lower than or equal to those for the 5-year screening interval, and the 7-4-1 screening protocol for those in the low, intermediate-low, and intermediate-high-risk categories performed best. The difference in the optimal protocols between our study and the UK study may be explained by the differences in duration of follow-up, risk threshold for high-risk, and reference protocols. Our study observed a shorter mean time spent in non-high-risk states (<10% risk, 15.8 years) compared to the UK study (<7.5% risk, 19.8 years^13^), possibly due to the latter's occupational cohort being healthier than the general population. The risk prediction models used in these two studies also differed in terms of whether laboratory test indicators were included. Nonetheless, using both hard and soft ASCVD outcomes, our study confirms that a risk-category-based screening strategy would enhance the cost-effectiveness of ASCVD primary prevention.

Due to differences in factors such as statin therapy costs and associated costs, ASCVD incidence, and absolute risk levels between countries, there are significant differences in the cost-effectiveness parameters of statin therapy for high-risk populations used in the above UK study and this study. The West of Scotland Coronary Prevention Study, which was referred to in the UK study, found that statin therapy for CVD prevention reduced costs in middle-aged men.^27^ While cost-effectiveness analyses based on the Chinese populations revealed incremental costs for primary prevention with statins^22^^,^^28^; these findings were consistent with those found in most other countries.^29^, ^30^, ^31^ Our study also suggests that if the incremental cost of statin therapy in China could be reduced by lowering treatment-related costs, such as purchasing drugs in primary care facilities and using risk assessment tools without blood lipid measurements, there would be more protocols that increase QALYs while saving costs.

In the current study, the 6-3-2 screening protocol would prevent approximately 1.95 more ASCVD events per 100,000 person-years than the uniform 3-yearly screening protocol, which accounts for about 0.57% of annual ASCVD incidence in China (342.17 per 100,000 person-years, according to the 2019 GBD study^21^). In comparison, using the 7-4-1 screening protocol in the 40–64 age group in England and Wales would prevent approximately 40.60 more ASCVD events per 100,000 person-years than the 5-yearly screening protocol (5034/12,400,000/100,000 person-years).^13^ One of the main reasons for the small number of ASCVD events avoided in our study could be due to the difference in the reference screening protocols used by the two studies, which were recommended by UK and Chinese guidelines, respectively, with our study using a more frequent screening protocol as a reference. Furthermore, the two studies differed in the source studies for determining the number of ASCVD events prevented by statins. The UK study referred to a cost-effectiveness analysis study that was electronically linked to the national hospitalization and death database to comprehensively record first and recurrent hospitalization events in cardiovascular disease, including MI, heart failure, stroke, coronary revascularization, and angiography, as well as hospitalization events for other coronary causes.^13^^,^^27^ The resulting ASCVD incidence is higher than in most other cost-effectiveness studies, which estimated the incidence using hard CVD outcomes and Markov model simulation. Our study relied on the latter as a parameter source.^22^

The CKB cohort used in this study included a large number of participants from diverse regions across China and with varying sociodemographic characteristics. The cohort maintains long-term follow-up on all participants, has an extremely low loss to follow-up rate, and ascertains as may study outcomes as possible in multiple ways. Regularly repeated surveys for some participants allow for the repeated measurements of CVD risk factors.

This study also has several limitations. First, the study population included individuals who participated in at least one resurvey, excluding those who died from cardiovascular events between baseline and resurveys. As a result, the absolute ASCVD risk level may be slightly lower than in the general population, potentially underestimating the transition probabilities to ASCVD events. To avoid the impact of survival bias, we used the ASCVD risk distribution from the CKB baseline population to estimate the number of people in different risk groups in the entire Chinese population. Second, the CKB-ASCVD models only used non-laboratory predictors in order to make them more accessible in a broader range of settings, especially in lower-resource regions. Because blood lipid information was not available for all participants in the baseline survey of the CKB study, we were unable to develop models with blood lipid variables or validate laboratory-based models that include blood lipid information in the CKB population. However, previous studies have found the WHO laboratory-based and non-laboratory-based models had similar predictive performances in the Chinese population.^17^ Third, we did not consider the additional benefits of initiating other interventions earlier, such as lifestyle modification antihypertensive and antidiabetic treatments, which could lead to underestimating the benefits of primary prevention in high-risk populations. However, current guidelines only recommend initiating statin therapy for primary prevention if there is an increased absolute risk of ASCVD. Antihypertensive and antidiabetic medications have not been widely used in populations at high risk of ASCVD but rather in those with hypertension and diabetes, respectively.

In this study of the Chinese population, risk-category-based screening interval strategies were found to be more cost-effective than a one-size-fits-all approach. All of the predictors in our risk assessment tool can be obtained from resident health records, which are part of the National Basic Public Health Service Program covering the entire Chinese population. Embedding the model for ASCVD risk assessment into an electronic health record system will facilitate risk-tailored screening and move the population-wide efforts toward more precision. Furthermore, our findings can help determine how frequently information in such a health record system needs to be updated.

## Contributors

JL conceived of and designed the paper. LL, ZC, and JC, as the members of the CKB steering committee, designed and supervised the CKB study, obtained funding, and, together with CY, DS, YP, PP, LY, YC, HD, HZ, XY, MB and RC, acquired the data. ZS did the analyses and YM verified the data. ZS wrote the first draft of the manuscript. JL helped to interpret the results. JL contributed to the critical revision of the manuscript for important intellectual content. All authors reviewed and approved the final manuscript. JL had access to raw data and had final responsibility for the decision to submit for publication.

## Data sharing statement

The access policy and procedures are available at www.ckbiobank.org.

## Declaration of interests

We declare no competing interests.
