# Supplementary material for: One-size-fits-all versus risk-category-based screening interval strategies for cardiovascular disease prevention in Chinese adults: a prospective cohort study
Source: Lancet Reg Health West Pac. 2024 Jul 16;49:101140. doi: 10.1016/j.lanwpc.2024.101140 (PMC11287009; doi:10.1016/j.lanwpc.2024.101140)
Supplement: Supplementary material [file mmc1.docx]

**One-size-fits-all versus risk-category-based screening interval strategies for cardiovascular disease prevention in Chinese adults: a prospective cohort study**

**Supplementary Online Content**

[Members of the China Kadoorie Biobank collaborative group 2](#_Toc167741221)

[Supplementary Methods 3](#_Toc167741222)

[eFigure 1. Calibration plots for CKB-ASCVD hard and soft outcome model after recalibration. 4](#_Toc167741223)

[eFigure 2. Observed and expected prevalence in each state 9](#_Toc167741224)

[eFigure 3. Estimated transition probability from low- and intermediate-risk category to high-risk category 10](#_Toc167741225)

[eFigure 4. Estimated mean sojourn time in each ASCVD 10-year-risk category and transition probabilities to the next risk category 13](#_Toc167741226)

[eFigure 5. Comparisons of all screening interval protocols with 3-yearly screening protocol with 10-year risk estimated by CKB-ASCVD soft outcome model 15](#_Toc167741227)

[eFigure 6. Comparisons of all screening interval protocols with 3-yearly screening protocol in men 17](#_Toc167741228)

[eFigure 7. Comparisons of all screening interval protocols with 3-yearly screening protocol in women 19](#_Toc167741229)

[eFigure 8. Comparisons of all screening interval protocols with 3-yearly screening protocol using the low limit of incremental cost of statin treatment 21](#_Toc167741230)

[eTable 1. Beta coefficients for predictor variables in CKB-ASCVD models 22](#_Toc167741231)

[eTable 2. Recalibration parameters for each region in CKB-ASCVD models 23](#_Toc167741232)

[eTable 3. Screening intervals for each screening protocol (years) 24](#_Toc167741233)

[eTable 4. Age-sex-specific 10-year risk distribution of the baseline population of CKB study (n=489,594) 25](#_Toc167741234)

[eTable 5. Age-sex-specific number of Chinese population free of ASCVD 26](#_Toc167741235)

[eTable 6. 10-year risk distribution of the study population with risk estimated by CKB-ASCVD soft outcome model 27](#_Toc167741236)

[eTable 7. Comparisons of all screening interval protocols with 3-yearly screening protocol with 10-year risk estimated by CKB-ASCVD hard outcome model 28](#_Toc167741237)

[eTable 8. Comparisons of all screening interval protocols with 3-yearly screening protocol with 10-year risk estimated by CKB-ASCVD soft outcome model 35](#_Toc167741238)

[eTable 9. Comparisons of all screening interval protocols with 3-yearly screening protocol using the low limit of incremental costs of statin treatment 42](#_Toc167741239)

**Members of the China Kadoorie Biobank collaborative group**

**International Steering Committee:** Junshi Chen, Zhengming Chen (PI), Robert Clarke, Rory Collins, Liming Li (PI), Jun Lv, Richard Peto, Robin Walters.

**International Co-ordinating Centre, Oxford:** Daniel Avery, Maxim Barnard, Derrick Bennett, Lazaros Belbasis, Ruth Boxall, Ka Hung Chan, Yiping Chen, Zhengming Chen, Charlotte Clarke, Johnathan Clarke; Robert Clarke, Huaidong Du, Ahmed Edris Mohamed, Hannah Fry, Simon Gilbert, Pek Kei Im, Andri Iona, Maria Kakkoura, Christiana Kartsonaki, Hubert Lam, Kuang Lin, James Liu, Mohsen Mazidi, Iona Millwood, Sam Morris, Qunhua Nie, Alfred Pozarickij, Maryanm Rahmati, Paul Ryder, Saredo Said, Dan Schmidt, Becky Stevens, Iain Turnbull, Robin Walters, Baihan Wang, Lin Wang, Neil Wright, Ling Yang, Xiaoming Yang, Pang Yao.

**National Co-ordinating Centre, Beijing:** Xiao Han, Can Hou, Qingmei Xia, Chao Liu, Jun Lv, Pei Pei, Dianjianyi Sun, Canqing Yu, Lang Pan.

**10 Regional Co-ordinating Centres:**

**Qingdao CDC:** Zengchang Pang, Ruqin Gao, Shanpeng Li, Haiping Duan, Shaojie Wang, Yongmei Liu, Ranran Du, Yajing Zang, Liang Cheng, Xiaocao Tian, Hua Zhang, Yaoming Zhai, Feng Ning, Xiaohui Sun, Feifei Li. **Licang CDC:** Silu Lv, Junzheng Wang, Wei Hou. **Heilongjiang Provincial CDC:** Wei Sun, Shichun Yan, Xiaoming Cui. **Nangang CDC:** Chi Wang, Zhenyuan Wu,Yanjie Li, Quan Kang. **Hainan Provincial CDC:** Huiming Luo, Tingting Ou. **Meilan CDC:** Xiangyang Zheng, Zhendong Guo, Shukuan Wu, Yilei Li, Huimei Li. **Jiangsu Provincial CDC:** Ming Wu, Yonglin Zhou, Jinyi Zhou, Ran Tao, Jie Yang, Jian Su. **Suzhou CDC:** Fang Liu, Jun Zhang, Yihe Hu, Yan Lu, Liangcai Ma, Aiyu Tang, Shuo Zhang, Jianrong Jin, Jingchao Liu. **Guangxi Provincial CDC:** Mei Lin, Zhenzhen Lu. **Liuzhou CDC:** Lifang Zhou, Changping Xie, Jian Lan,Tingping Zhu,Yun Liu, Liuping Wei, Liyuan Zhou, Ningyu Chen, Yulu Qin, Sisi Wang. **Sichuan Provincial CDC:** Xianping Wu, Ningmei Zhang, Xiaofang Chen, Xiaoyu Chang. **Pengzhou CDC:** Mingqiang Yuan, Xia Wu, Xiaofang Chen, Wei Jiang, Jiaqiu Liu, Qiang Sun. **Gansu Provincial CDC:** Faqing Chen, Xiaolan Ren, Caixia Dong. **Maiji CDC:** Hui Zhang, Enke Mao, Xiaoping Wang, Tao Wang, Xi zhang. **Henan Provincial CDC:** Kai Kang, Shixian Feng, Huizi Tian, Lei Fan. **Huixian CDC:** XiaoLin Li, Huarong Sun, Pan He, Xukui Zhang. **Zhejiang Provincial CDC:** Min Yu, Ruying Hu, Hao Wang. **Tongxiang CDC**: Xiaoyi Zhang, Yuan Cao, Kaixu Xie, Lingli Chen, Dun Shen. **Hunan Provincial CDC:** Xiaojun Li, Donghui Jin, Li Yin, Huilin Liu, Zhongxi Fu. **Liuyang CDC:** Xin Xu, Hao Zhang, Jianwei Chen,Yuan Peng, Libo Zhang, Chan Qu.

# Supplementary Methods

The detailed process of model construction has been described previously.^1^ The following is a brief introduction to the calculation of 10-year risk of cardiovascular disease:

Step 1: Calculate the linear predictors using the beta coefficients in the eTable 1:

$$lp=\beta_{age}\times\left( age-55 \right)+\beta_{sbp}\times\left( sbp-120 \right)+\beta_{dbp}\times\left( dbp-80 \right)+\beta_{hpt}\times hpt+\beta_{sms}\times sms+\beta_{dia}\times dia+\beta_{wai}\times\left( wai-80 \right)+\beta_{aeg\times sbp}\times age\times sbp+\beta_{aeg\times dbp}\times age\times dbp+\beta_{aeg\times hpt}\times age\times hpt+\beta_{aeg\times sms}\times age\times sms+\beta_{aeg\times dia}\times age\times dia+\beta_{aeg\times wai}\times age\times wai$$

Step 2: Calculate the originally predicted 10-year risk using the linear predictors and baseline survival estimate at 10 years for each outcome:

$$risk=1-S^{exp(lp)}$$

Step 3: Calculate the recalibrated 10-year risk (R) for each outcome ($R_{IHD}, R_{IS}{, R}_{hard-ASCVD})$using the recalibration parameters in the eTable 2:

$$R=1 - \exp(-\exp(b + k \times\ln(-\ln(1 - \mathrm{risk}))))$$

The predicted 10-year risk for hard ASCVD outcome was $R_{hard-ASCVD}$. The predicted 10-year risk for soft ASCVD outcome was calculated as the following formula:

$$R_{soft-ASCVD}=1-(1-R_{IHD}) \times(1-R_{IS})$$

Reference:

1. Yang S, Han Y, Yu C, et al. Development of a Model to Predict 10-Year Risk of Ischemic and Hemorrhagic Stroke and Ischemic Heart Disease Using the China Kadoorie Biobank. *Neurology* 2022; **98**(23): e2307-e17.


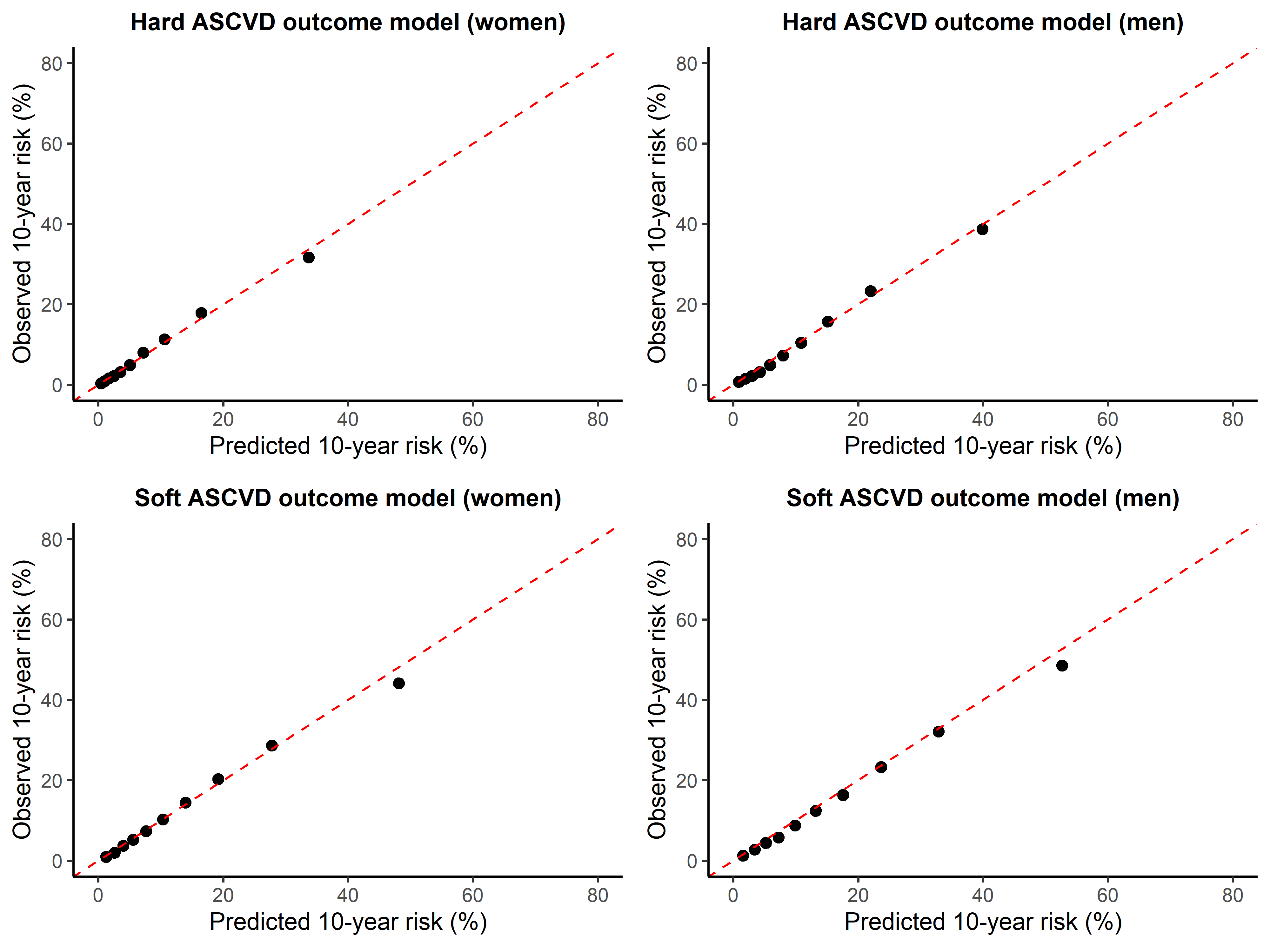


# eFigure 1. Calibration plots for CKB-ASCVD hard and soft outcome model after recalibration.

CKB: China Kadoorie Biobank; ASCVD: atherosclerotic cardiovascular disease.

(A)


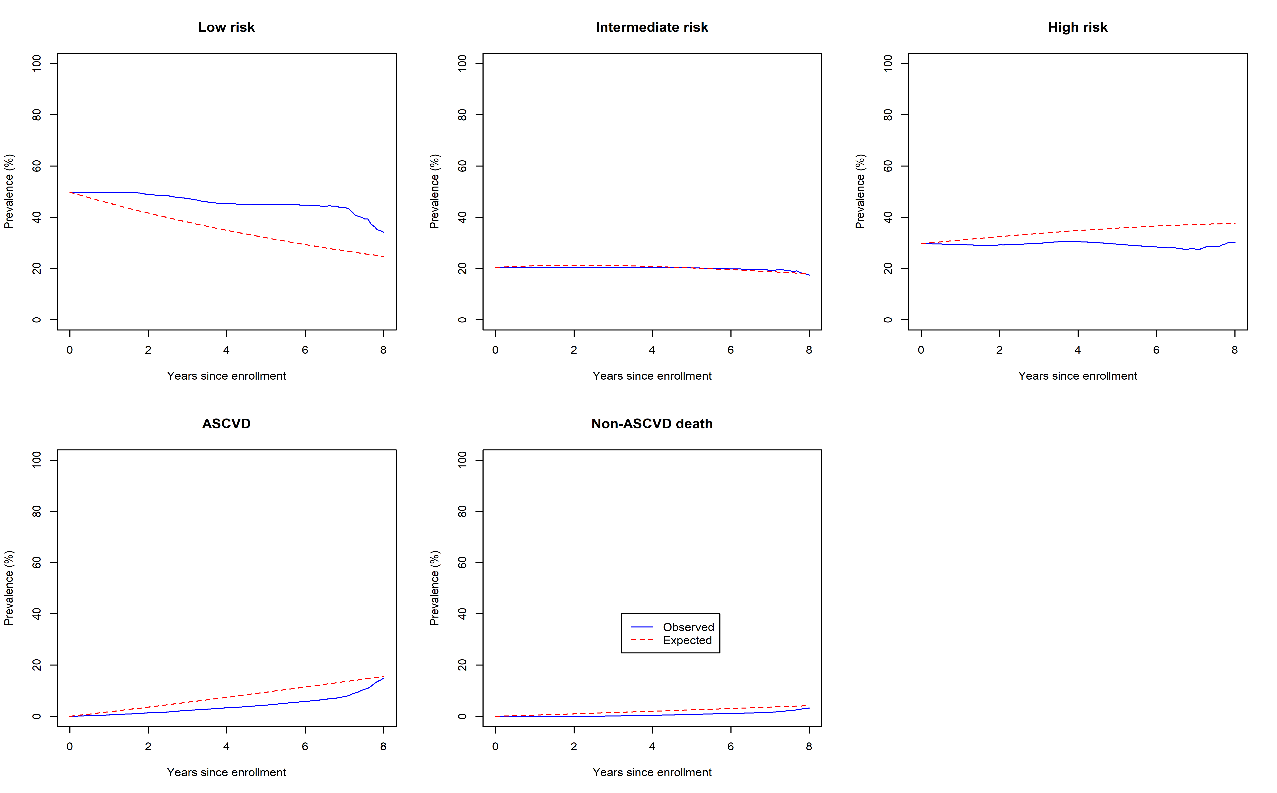


(B)


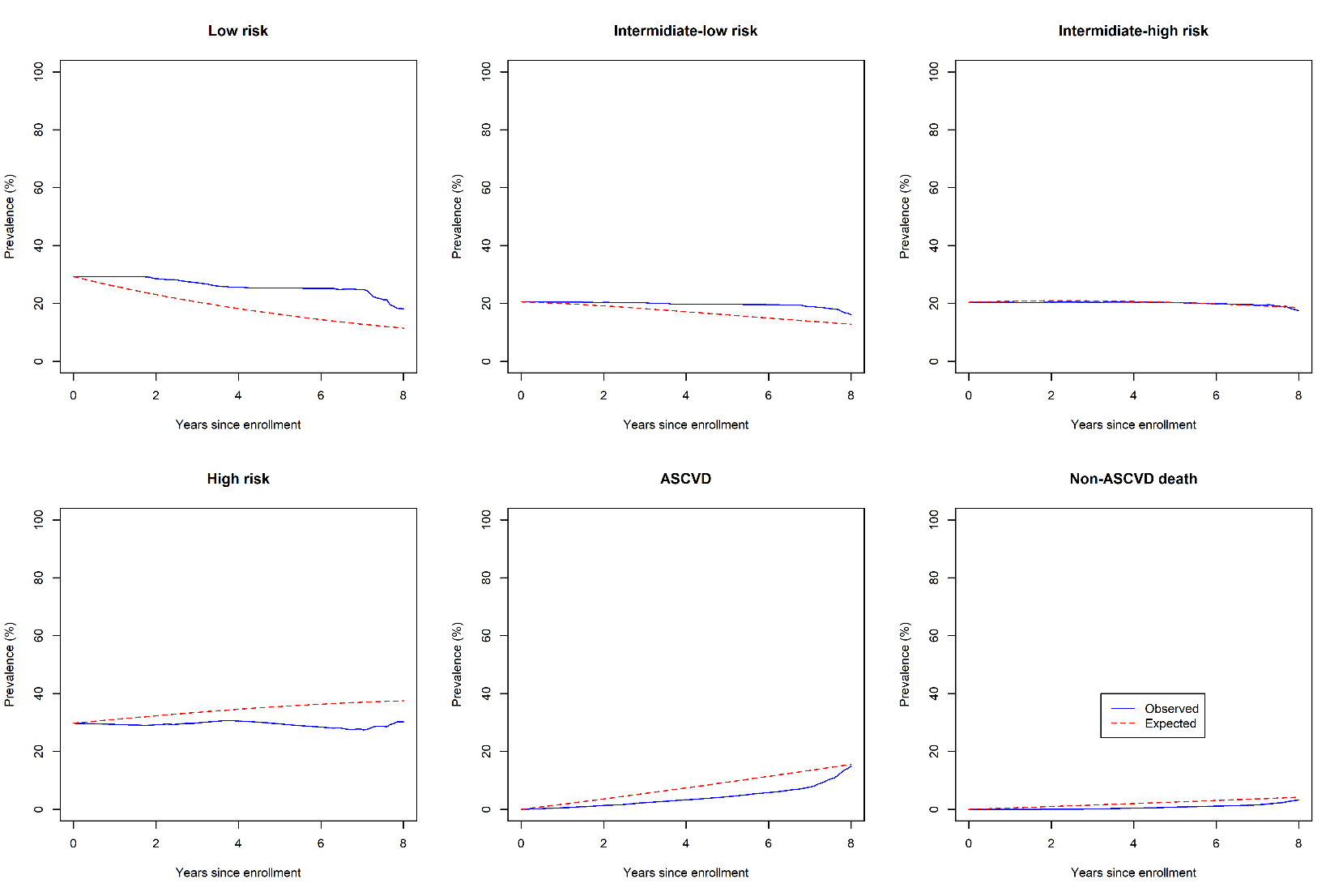


(C)


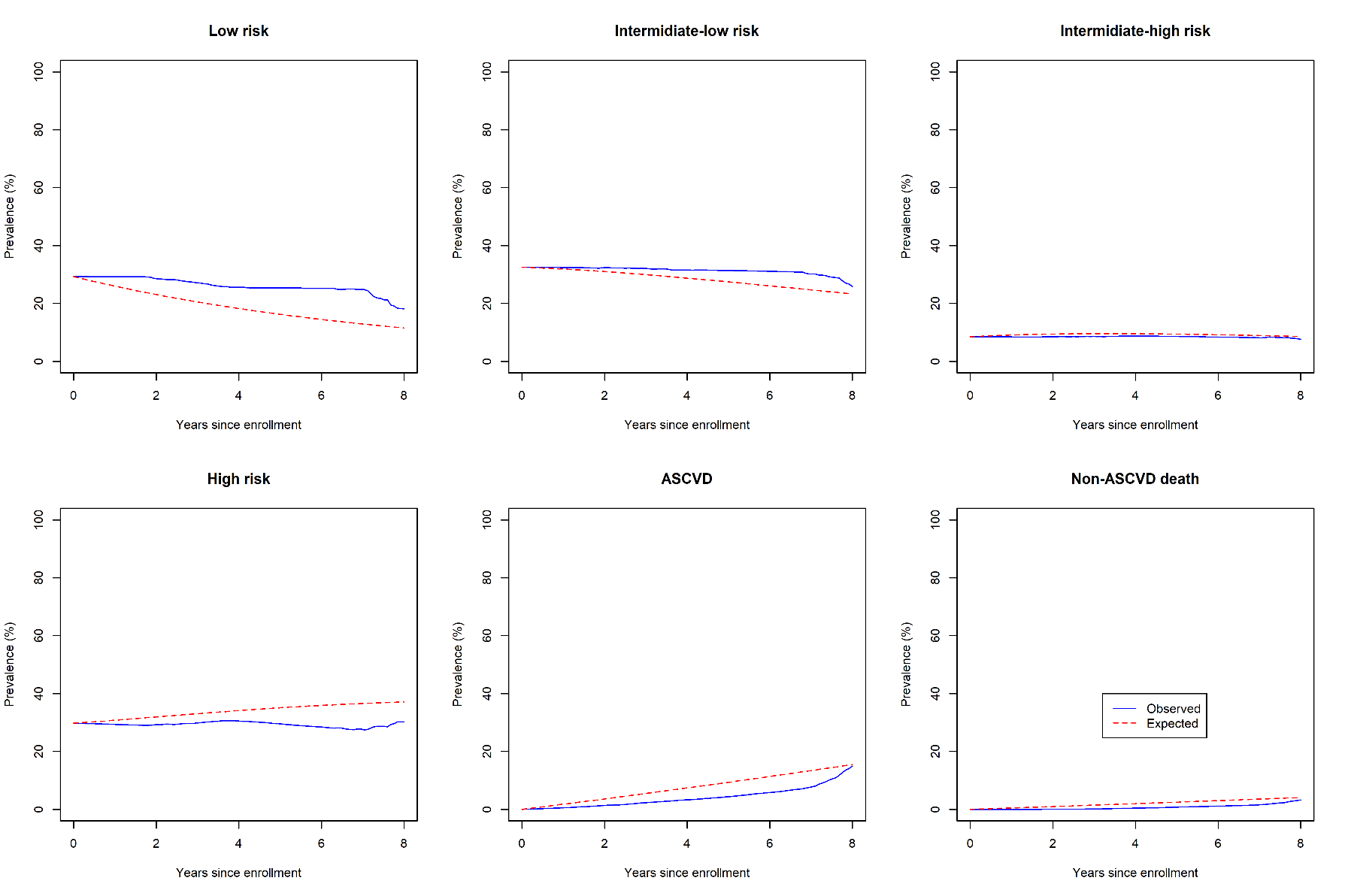


(D)


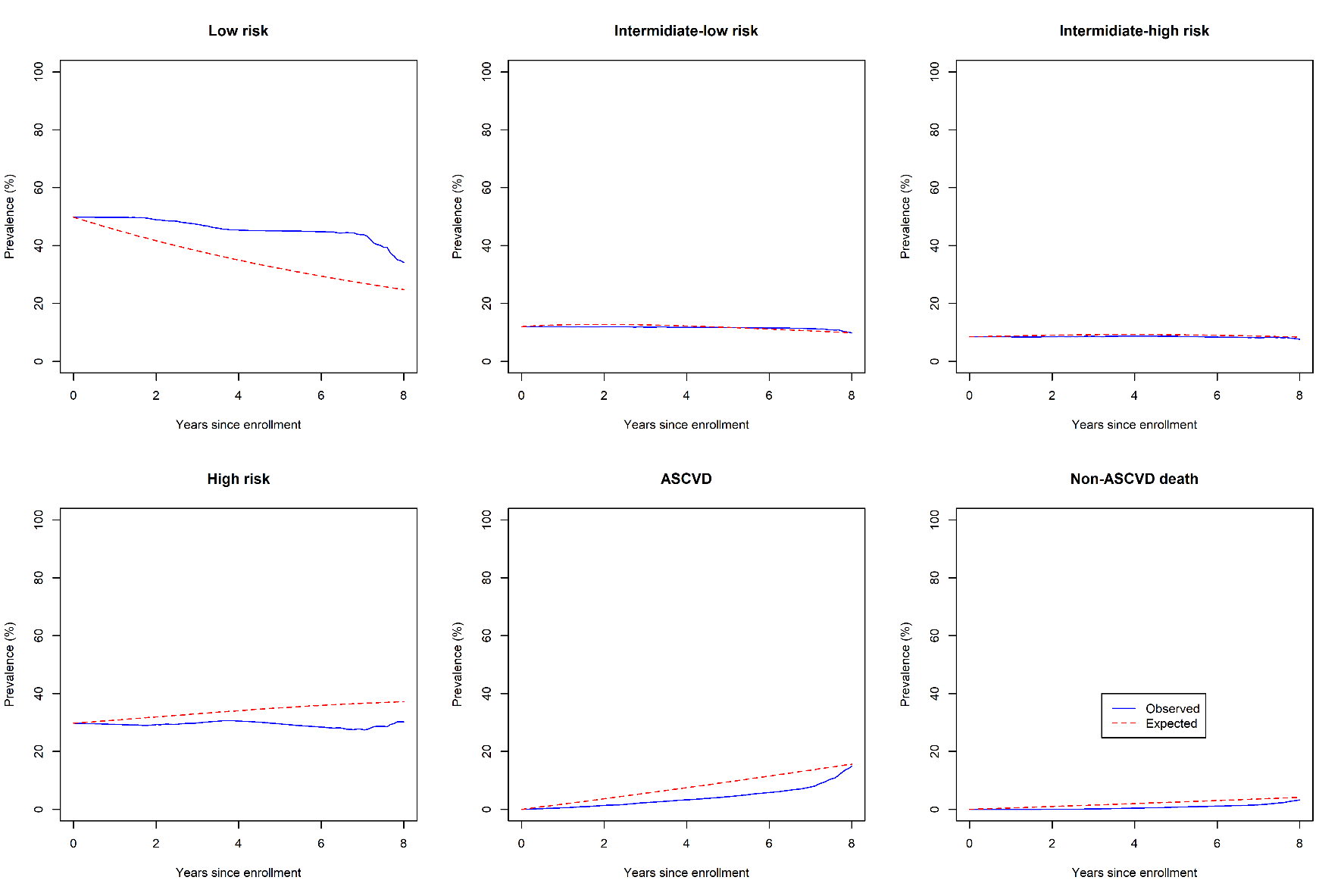


# eFigure 2. Observed and expected prevalence in each state

ASCVD: atherosclerotic cardiovascular disease.

Blue and red lines indicate the observed and expected prevalence, respectively. Times on the x-axis indicate years. The risk cut-offs were different in the four figures, namely:

(A) low (<5.0%), intermediate (5.0%-9.9%), high risk (≥10.0%);

(B) low (<2.5%), intermediate-low (2.5%-4.9%), intermediate-high (5.0%-9.9%), and high risk (≥10.0%);

(C) low (<2.5%), intermediate-low (2.5%-7.4%), intermediate-high (7.5%-9.9%), and high risk (≥10.0%);

(D) low (<5.0%), intermediate-low (5.0%-7.4%), intermediate-high (7.5%-9.9%), and high risk (≥10.0%).


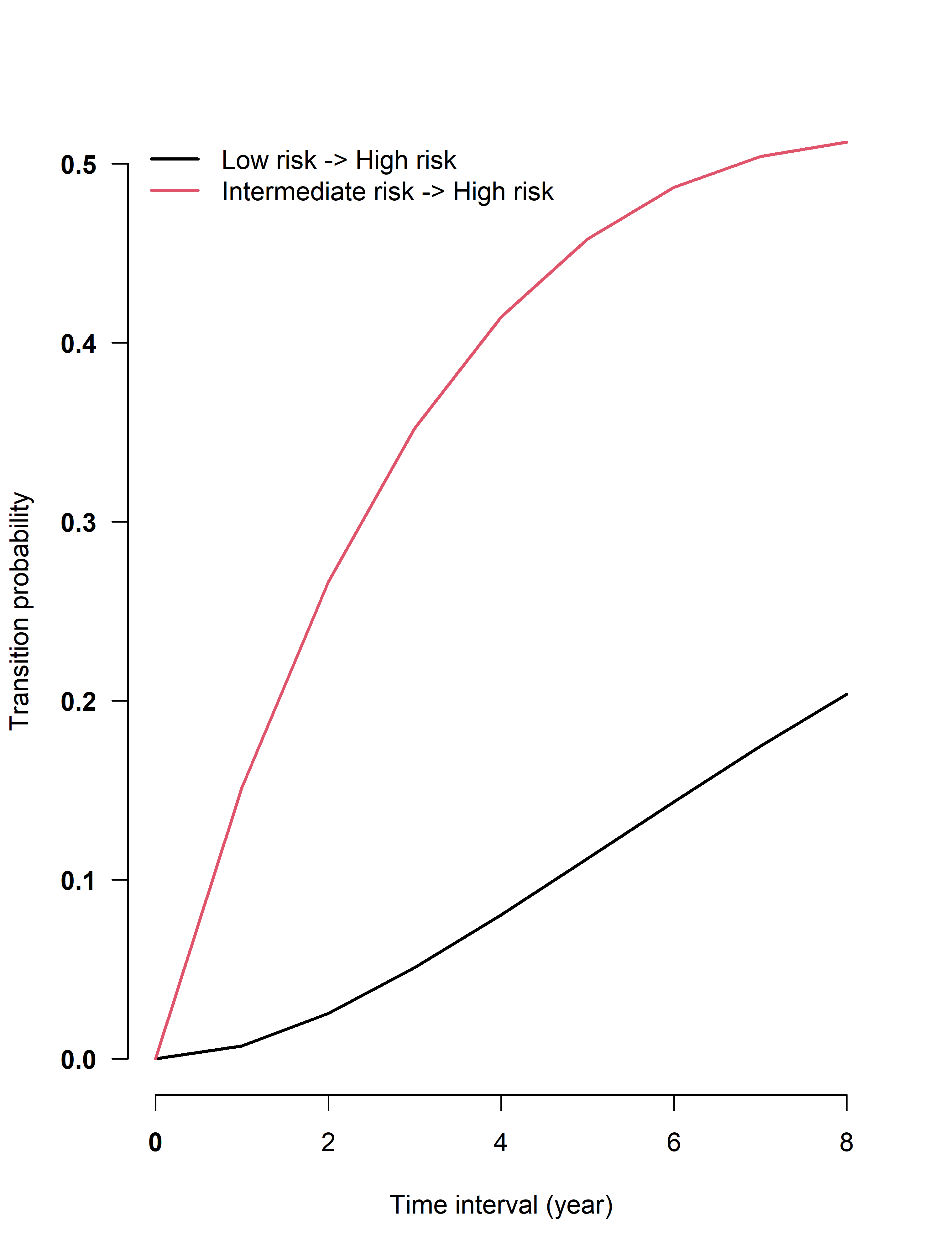


# eFigure 3. Estimated transition probability from low- and intermediate-risk category to high-risk category

The 10-year risks were estimated by CKB-ASCVD hard outcome model. Risk categories were defined as low (<5.0%), intermediate (5.0%-9.9%), and high risk (≥10.0%).

(A)


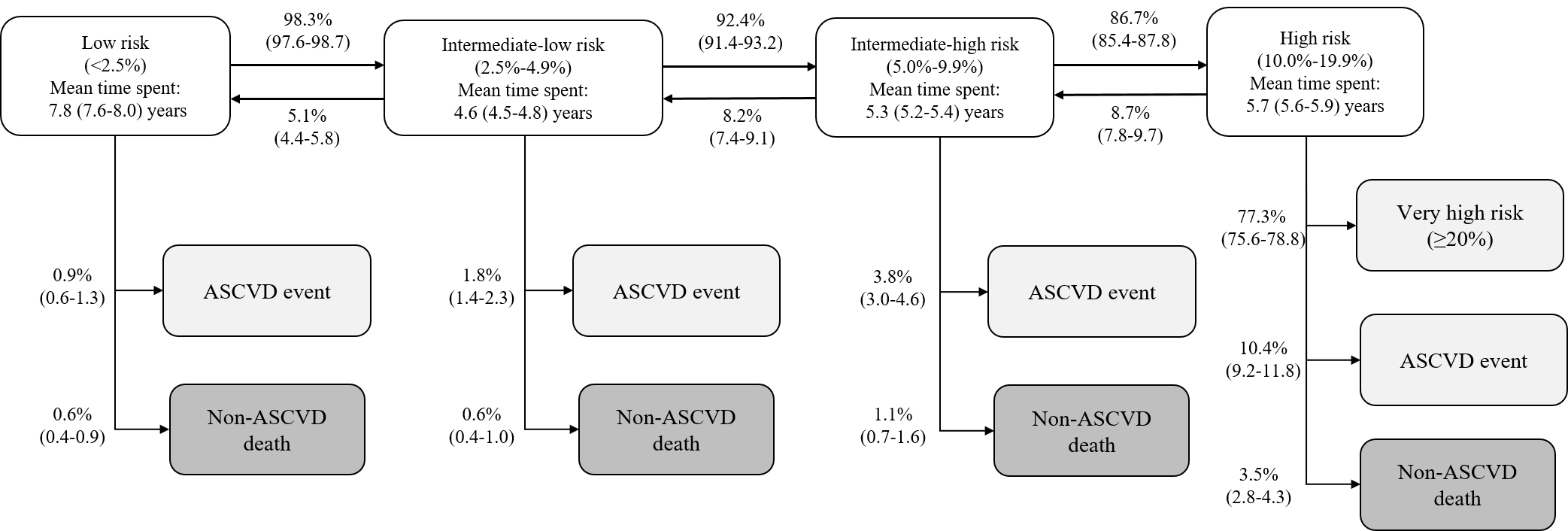


(B)


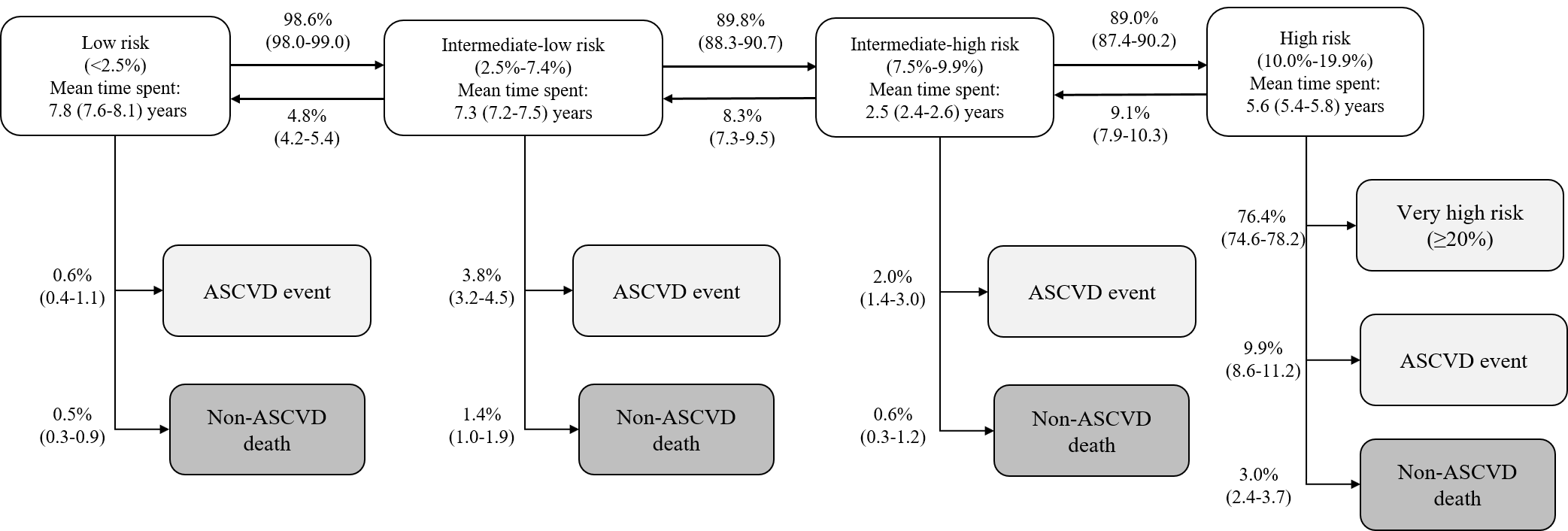


(C)


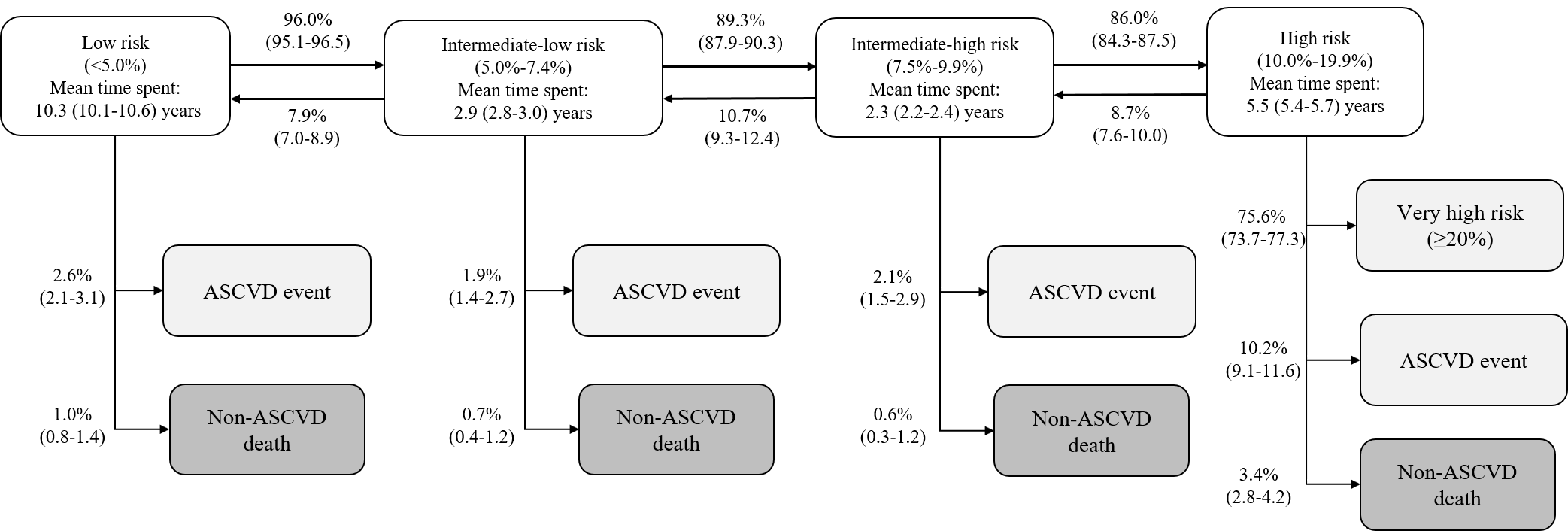


# eFigure 4. Estimated mean sojourn time in each ASCVD 10-year-risk category and transition probabilities to the next risk category

ASCVD: atherosclerotic cardiovascular disease.

Data are estimates with 95% CIs. The 10-year risks were estimated by CKB-ASCVD hard outcome model.

The risk cut-offs were different in the three figures, namely:

(A) low (<2.5%), intermediate-low (2.5%-4.9%), intermediate-high (5.0%-9.9%);

(B) low (<2.5%), intermediate-low (2.5%-7.4%), intermediate-high (7.5%-9.9%);

(C) low (<5.0%), intermediate-low (5.0%-7.4%), intermediate-high (7.5%-9.9%).


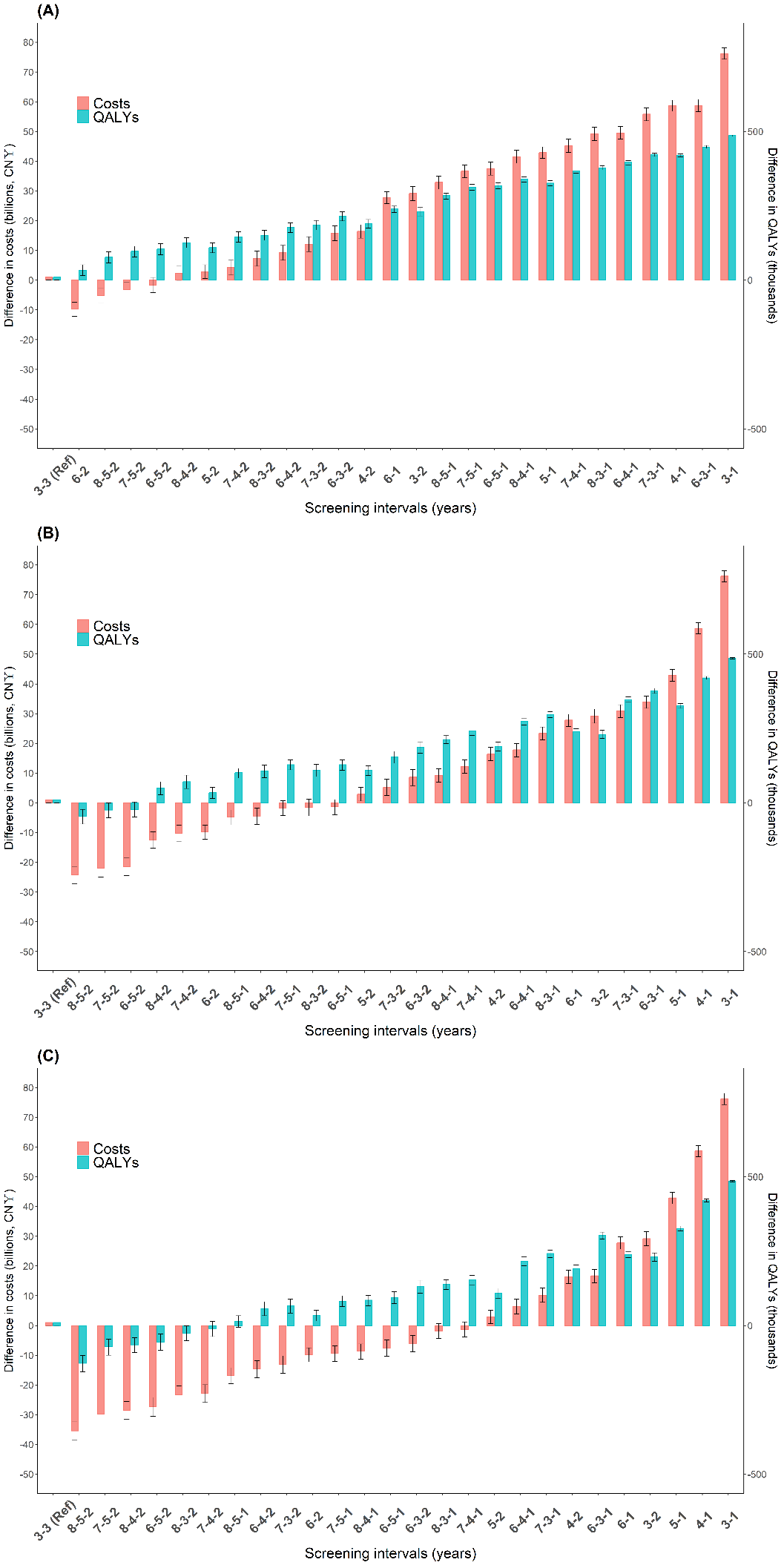


# eFigure 5. Comparisons of all screening interval protocols with 3-yearly screening protocol with 10-year risk estimated by CKB-ASCVD soft outcome model

QALY: quality-adjusted life year.

Red bars represent differences in total health-care costs, and green bars represent differences in QALYs gained in the Chinese population over a 10-year period. Data are estimates with 95% CIs. In the three figures, three-risk categories were uniformly defined as low (<10.0%), intermediate (10.0%-19.9%), and high risk (≥20.0%). The risk cut-offs of the four-risk categories were different in the three figures, namely: (A) low (<5.0%), intermediate-low (5.0%-9.9%), intermediate-high (10.0%-19.9%), and high risk (≥20.0%); (B) low (<5.0%), intermediate-low (5.0%-14.9%), intermediate-high (15.0%-19.9%), and high risk (≥20.0%); (C) low (<10.0%), intermediate-low (10.0%-14.9%), intermediate-high (15.0%-19.9%), and high risk (≥20.0%).


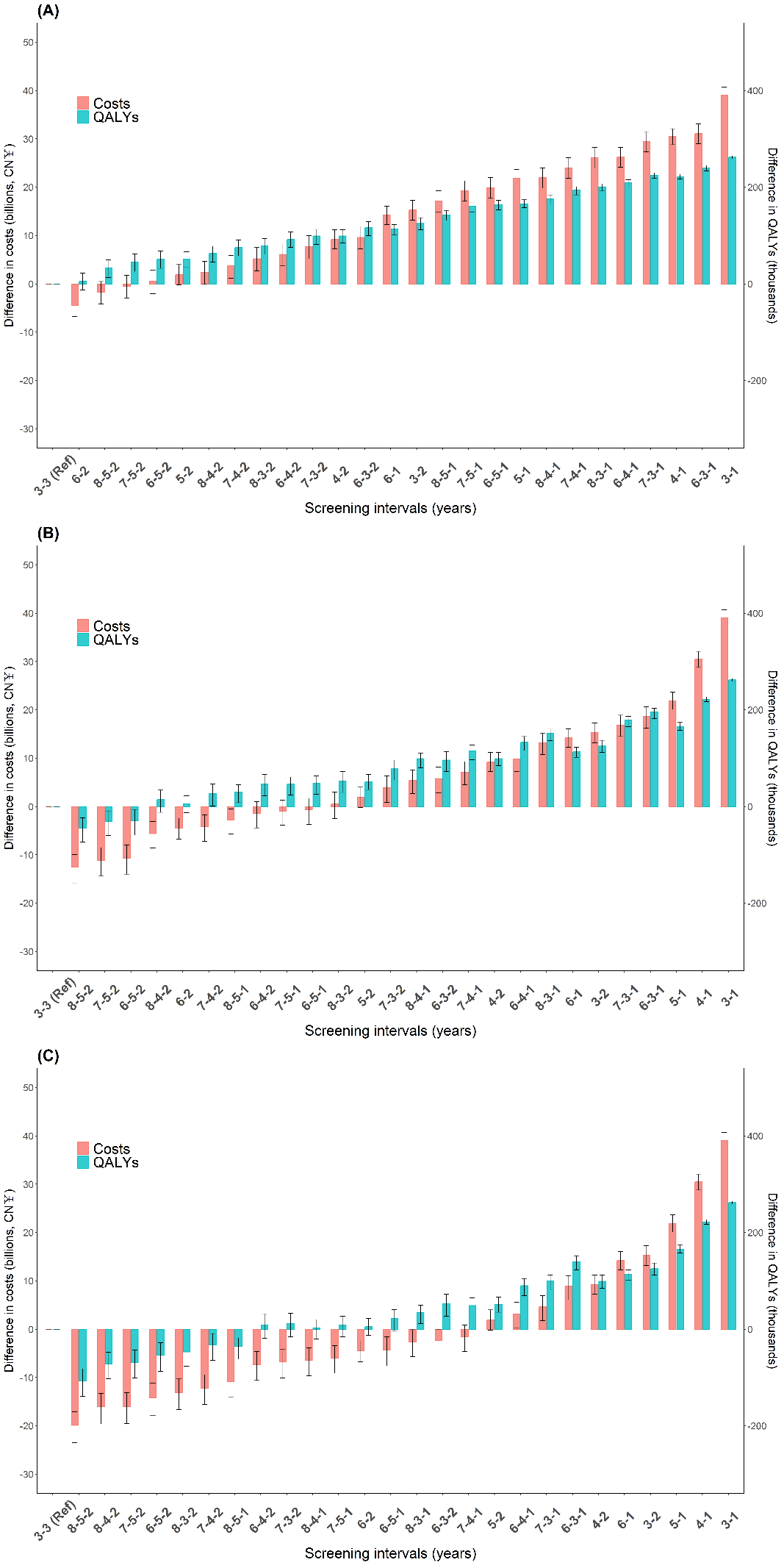


# eFigure 6. Comparisons of all screening interval protocols with 3-yearly screening protocol in men

QALY: quality-adjusted life year.

Red bars represent differences in total health-care costs, and green bars represent differences in QALYs gained in the Chinese population over a 10-year period. Data are estimates with 95% CIs. The 10-year risks were estimated by CKB-ASCVD hard outcome model. In the three figures, three-risk categories were uniformly defined as low (<5.0%), intermediate (5.0%-9.9%), and high risk (≥10.0%). The risk cut-offs of the four-risk categories were different in the three figures, namely: (A) low (<2.5%), intermediate-low (2.5%-4.9%), intermediate-high (5.0%-9.9%), and high risk (≥10.0%); (B) low (<2.5%), intermediate-low (2.5%-7.4%), intermediate-high (7.5%-9.9%), and high risk (≥10.0%); (C) low (<5.0%), intermediate-low (5.0%-7.4%), intermediate-high (7.5%-9.9%), and high risk (≥10.0%).


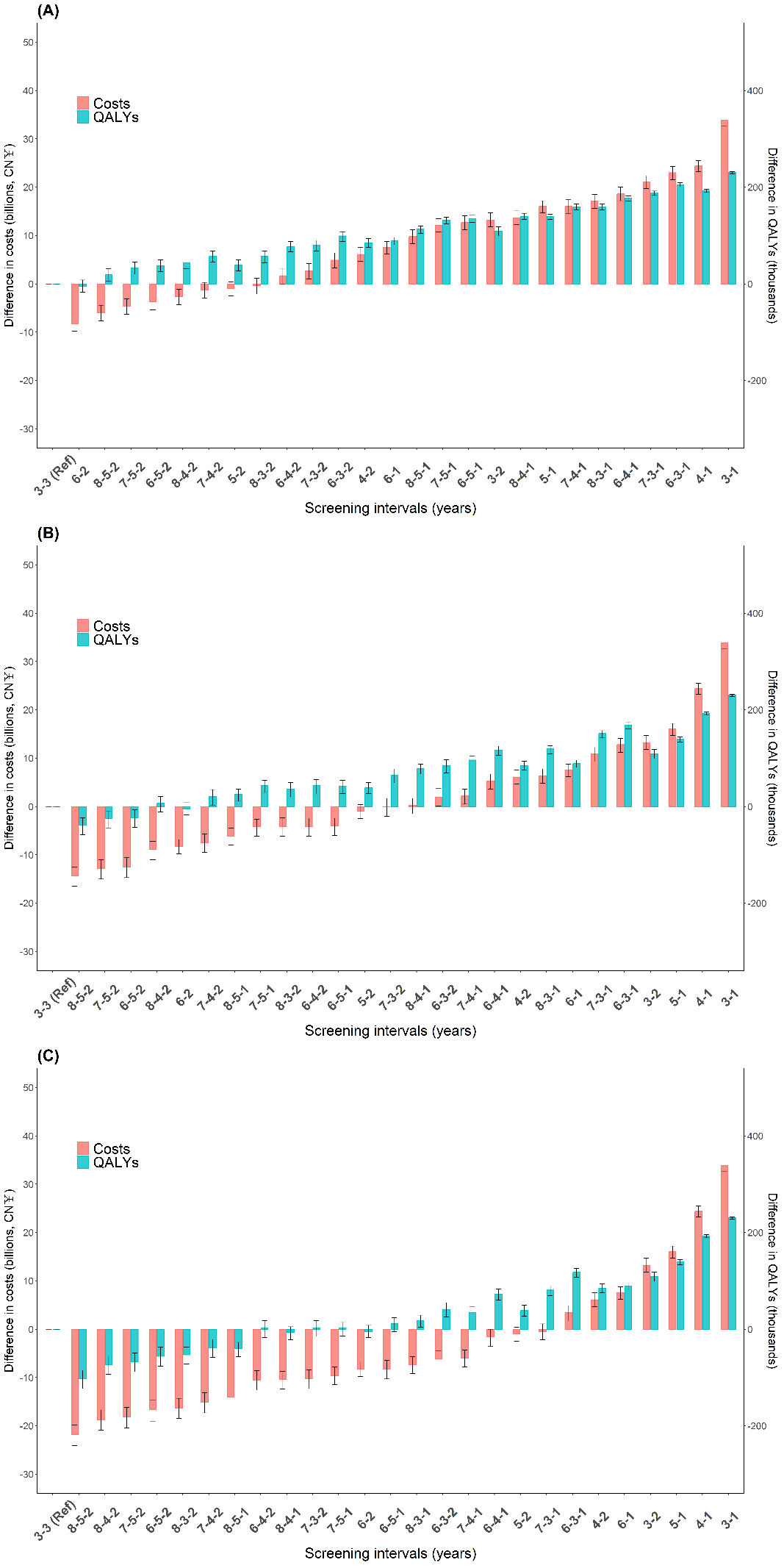


# eFigure 7. Comparisons of all screening interval protocols with 3-yearly screening protocol in women

QALY: quality-adjusted life year.

Red bars represent differences in total health-care costs, and green bars represent differences in QALYs gained in the Chinese population over a 10-year period. Data are estimates with 95% CIs. The 10-year risks were estimated by CKB-ASCVD hard outcome model. In the three figures, three-risk categories were uniformly defined as low (<5.0%), intermediate (5.0%-9.9%), and high risk (≥10.0%). The risk cut-offs of the four-risk categories were different in the three figures, namely: (A) low (<2.5%), intermediate-low (2.5%-4.9%), intermediate-high (5.0%-9.9%), and high risk (≥10.0%); (B) low (<2.5%), intermediate-low (2.5%-7.4%), intermediate-high (7.5%-9.9%), and high risk (≥10.0%); (C) low (<5.0%), intermediate-low (5.0%-7.4%), intermediate-high (7.5%-9.9%), and high risk (≥10.0%).


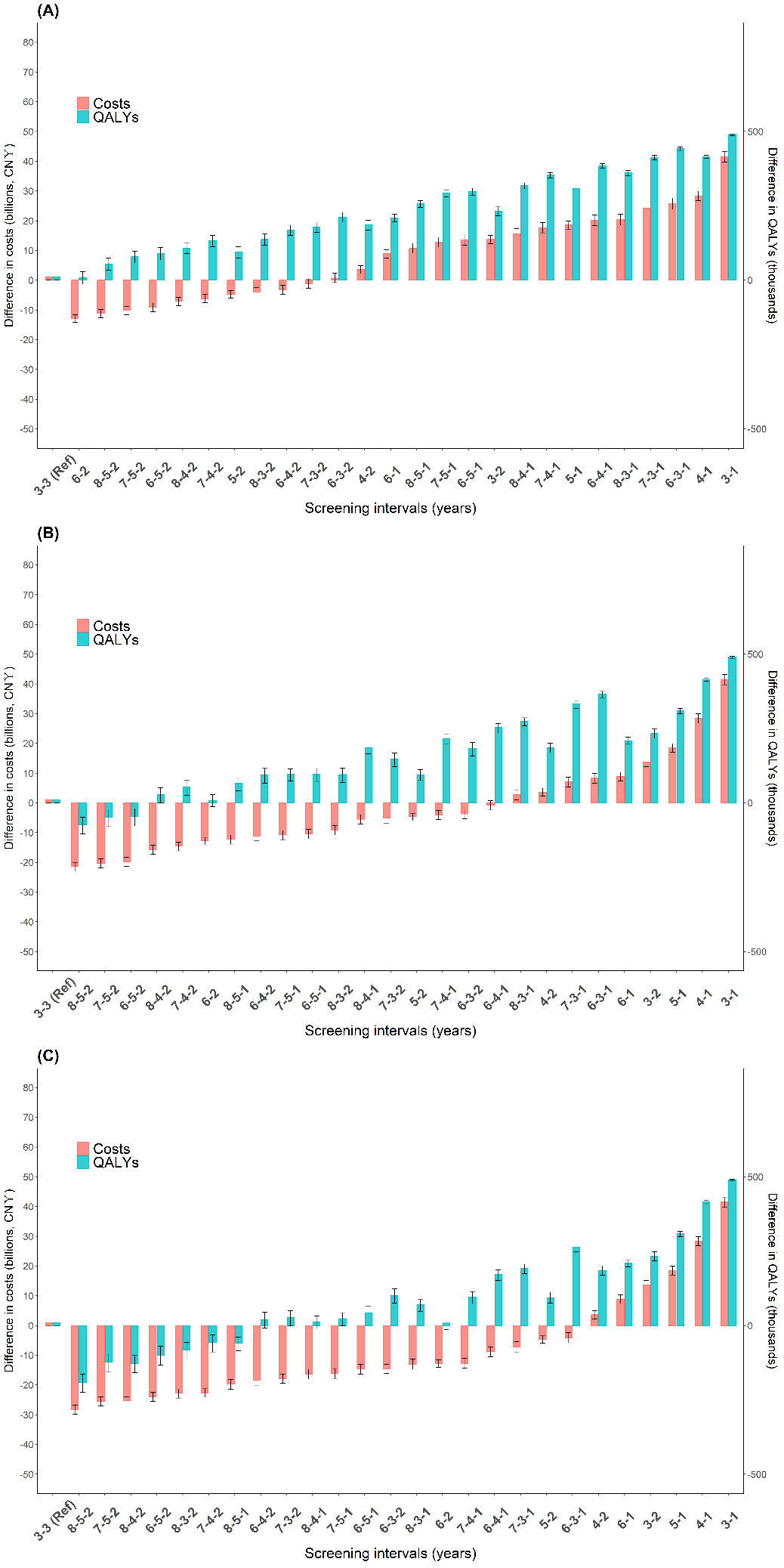


# eFigure 8. Comparisons of all screening interval protocols with 3-yearly screening protocol using the low limit of incremental cost of statin treatment

QALY: quality-adjusted life year.

Red bars represent differences in total health-care costs, and green bars represent differences in QALYs gained in the Chinese population over a 10-year period. Data are estimates with 95% CIs. The 10-year risks were estimated by CKB-ASCVD hard outcome model. In the three figures, three-risk categories were uniformly defined as low (<5.0%), intermediate (5.0%-9.9%), and high risk (≥10.0%). The risk cut-offs of the four-risk categories were different in the three figures, namely: (A) low (<2.5%), intermediate-low (2.5%-4.9%), intermediate-high (5.0%-9.9%), and high risk (≥10.0%); (B) low (<2.5%), intermediate-low (2.5%-7.4%), intermediate-high (7.5%-9.9%), and high risk (≥10.0%); (C) low (<5.0%), intermediate-low (5.0%-7.4%), intermediate-high (7.5%-9.9%), and high risk (≥10.0%).

# eTable 1. Beta coefficients for predictor variables in CKB-ASCVD models

| Predictor | Women | | |  | Men | | |
| --- | --- | --- | --- | --- | --- | --- | --- |
|  | IHD | IS | Hard-ASCVD |  | IHD | IS | Hard-ASCVD |
| Age (age), years | 0.067 | 0.074 | 0.077 |  | 0.072 | 0.084 | 0.086 |
| Systolic blood pressure (sbp), mmHg | 0.002 | 0.009 | 0.009 |  | 0.005 | 0.012 | 0.012 |
| Diastolic blood pressure (dbp), mmHg | 0.005 | 0.010 | 0.011 |  | 0.009 | 0.014 | 0.014 |
| Use of blood pressure-lowering treatment (hpt) | 0.438 | 0.432 | 0.426 |  | 0.466 | 0.466 | 0.464 |
| Current daily smoker (sms) | 0.160 | 0.259 | 0.304 |  | 0.202 | 0.251 | 0.286 |
| Self-reported diabetes (dia) | 0.463 | 0.599 | 0.658 |  | 0.597 | 0.606 | 0.636 |
| Waist circumference (wai), cm | 0.015 | 0.010 | 0.010 |  | 0.013 | 0.008 | 0.007 |
| age x sbp | 0.000 | 0.000 | 0.000 |  | 0.000 | 0.000 | 0.000 |
| age x dbp | 0.000 | 0.000 | 0.000 |  | -0.001 | 0.000 | 0.000 |
| age x hpt | -0.010 | -0.009 | -0.009 |  | -0.005 | -0.012 | -0.012 |
| age x sms | -0.007 | -0.016 | -0.012 |  | -0.004 | -0.005 | -0.006 |
| age x dia | -0.012 | -0.021 | -0.019 |  | -0.018 | -0.013 | -0.013 |
| age x wai | -0.001 | 0.000 | 0.000 |  | -0.001 | 0.000 | 0.000 |
| Baseline survival estimate at 10 years (S) | 0.900 | 0.910 | 0.906 |  | 0.929 | 0.928 | 0.920 |

CKB: China Kadoorie Biobank; ASCVD: atherosclerotic cardiovascular disease; IHD: ischemic heart disease; IS: ischemic stroke.

# eTable 2. Recalibration parameters for each region in CKB-ASCVD models

| Region | IHD | |  | IS | |  | Hard-ASCVD | |
| --- | --- | --- | --- | --- | --- | --- | --- | --- |
|  | k | b |  | k | b |  | k | b |
| Women |  |  |  |  |  |  |  |  |
| Qingdao | 1.204 | 0.247 |  | 1.377 | -0.448 |  | 1.332 | -0.351 |
| Harbin | 0.784 | 0.157 |  | 0.958 | 0.470 |  | 0.944 | 0.464 |
| Haikou | 1.120 | -0.463 |  | 1.072 | 0.291 |  | 1.034 | 0.239 |
| Suzhou | 1.448 | -1.124 |  | 1.318 | -0.765 |  | 1.235 | -0.999 |
| Liuzhou | 1.047 | -0.416 |  | 1.268 | -0.206 |  | 1.238 | -0.248 |
| Chengdu | 1.340 | 0.344 |  | 1.344 | -0.293 |  | 1.339 | -0.224 |
| Tianshui | 0.975 | -0.325 |  | 0.813 | -0.975 |  | 0.910 | -0.469 |
| Xinxiang | 1.160 | 0.195 |  | 1.207 | 0.417 |  | 1.174 | 0.379 |
| Jiaxing | 0.987 | -0.985 |  | 1.479 | -0.411 |  | 1.356 | -0.657 |
| Changsha | 0.903 | -0.359 |  | 0.856 | -0.778 |  | 0.863 | -0.611 |
| Men |  |  |  |  |  |  |  |  |
| Qingdao | 1.286 | 0.475 |  | 1.303 | -0.274 |  | 1.177 | -0.182 |
| Harbin | 0.732 | 0.034 |  | 0.911 | 0.432 |  | 0.906 | 0.431 |
| Haikou | 0.895 | -0.653 |  | 0.999 | 0.065 |  | 0.886 | -0.092 |
| Suzhou | 1.138 | -1.195 |  | 1.359 | -0.452 |  | 1.233 | -0.737 |
| Liuzhou | 0.956 | -0.354 |  | 0.761 | -0.716 |  | 0.749 | -0.706 |
| Chengdu | 1.395 | 0.347 |  | 1.400 | -0.250 |  | 1.231 | -0.479 |
| Tianshui | 0.886 | -0.188 |  | 0.818 | -0.972 |  | 0.941 | -0.392 |
| Xinxiang | 1.068 | 0.039 |  | 1.038 | 0.238 |  | 1.042 | 0.259 |
| Jiaxing | 1.123 | -0.562 |  | 0.937 | -1.090 |  | 0.878 | -1.186 |
| Changsha | 0.924 | 0.062 |  | 0.831 | -0.627 |  | 0.828 | -0.417 |

CKB: China Kadoorie Biobank; ASCVD: atherosclerotic cardiovascular disease; IHD: ischemic heart disease; IS: ischemic stroke.

# eTable 3. Screening intervals for each screening protocol (years)

| Three risk categories | | |
| --- | --- | --- |
| Low-risk | Intermediate-risk | |
| 3 | 3 | |
| 3 | 1 | |
| 3 | 2 | |
| 4 | 1 | |
| 4 | 2 | |
| 5 | 1 | |
| 5 | 2 | |
| 6 | 1 | |
| 6 | 2 | |
| Four risk categories | | |
| Low-risk | Intermediate-low-risk | Intermediate-high-risk |
| 6 | 3 | 1 |
| 6 | 3 | 2 |
| 6 | 4 | 1 |
| 6 | 4 | 2 |
| 6 | 5 | 1 |
| 6 | 5 | 2 |
| 7 | 3 | 1 |
| 7 | 3 | 2 |
| 7 | 4 | 1 |
| 7 | 4 | 2 |
| 7 | 5 | 1 |
| 7 | 5 | 2 |
| 8 | 3 | 1 |
| 8 | 3 | 2 |
| 8 | 4 | 1 |
| 8 | 4 | 2 |
| 8 | 5 | 1 |
| 8 | 5 | 2 |

# eTable 4. Age-sex-specific 10-year risk distribution of the baseline population of CKB study (n=489,594)

|  | Risks estimated by CKB-ASCVD hard outcome model | | | | |  | Risks estimated by CKB-ASCVD soft outcome model | | | | |
| --- | --- | --- | --- | --- | --- | --- | --- | --- | --- | --- | --- |
|  | <2.5% | 2.5%-4.9% | 5.0%-7.4% | 7.5%-9.9% | ≥10.0% |  | <5.0% | 5.0%-9.9% | 10.0%-14.9% | 15.0%-19.9% | ≥20.0% |
| Men |  |  |  |  |  |  |  |  |  |  |  |
| 30-34 | 69.1 | 21.9 | 5.6 | 1.7 | 1.8 |  | 70.2 | 19.5 | 7.4 | 1.7 | 1.2 |
| 35-39 | 61.4 | 27.4 | 6.3 | 2.5 | 2.5 |  | 68.3 | 22.3 | 5.5 | 2.2 | 1.7 |
| 40-44 | 40.1 | 34.2 | 12.9 | 5.5 | 7.3 |  | 46.3 | 34.2 | 9.7 | 4.7 | 5.0 |
| 45-49 | 20.8 | 30.2 | 20.9 | 10.2 | 17.9 |  | 26.2 | 35.5 | 18.0 | 7.9 | 12.5 |
| 50-54 | 9.9 | 24.4 | 22.0 | 15.9 | 27.9 |  | 15.1 | 27.7 | 26.9 | 12.4 | 18.0 |
| 55-59 | 1.8 | 14.8 | 16.6 | 17.1 | 49.8 |  | 4.0 | 19.2 | 21.8 | 22.2 | 32.8 |
| 60-64 | 0.0 | 3.6 | 11.5 | 12.2 | 72.7 |  | 0.4 | 7.7 | 14.2 | 17.3 | 60.5 |
| 65-69 | 0.0 | 0.1 | 2.0 | 6.4 | 91.5 |  | 0.0 | 0.8 | 5.2 | 8.8 | 85.3 |
| 70-74 | 0.0 | 0.0 | 0.0 | 0.5 | 99.5 |  | 0.0 | 0.0 | 0.5 | 2.9 | 96.7 |
| 75-79 | 0.0 | 0.0 | 0.0 | 0.0 | 100.0 |  | 0.0 | 0.0 | 0.0 | 0.1 | 99.9 |
| Women |  |  |  |  |  |  |  |  |  |  |  |
| 30-34 | 89.7 | 8.9 | 0.8 | 0.3 | 0.3 |  | 82.0 | 15.8 | 1.8 | 0.3 | 0.2 |
| 35-39 | 82.7 | 14.2 | 2.1 | 0.5 | 0.5 |  | 80.9 | 14.6 | 3.5 | 0.6 | 0.3 |
| 40-44 | 59.8 | 27.8 | 7.5 | 2.5 | 2.4 |  | 57.4 | 28.8 | 8.6 | 3.4 | 1.9 |
| 45-49 | 40.2 | 26.4 | 15.9 | 8.1 | 9.4 |  | 31.4 | 38.0 | 13.6 | 8.5 | 8.7 |
| 50-54 | 24.4 | 25.0 | 18.7 | 11.4 | 20.4 |  | 15.7 | 32.5 | 25.4 | 9.6 | 16.9 |
| 55-59 | 7.0 | 24.0 | 17.9 | 14.7 | 36.5 |  | 6.4 | 18.6 | 26.1 | 20.0 | 28.9 |
| 60-64 | 0.2 | 10.7 | 15.4 | 14.2 | 59.6 |  | 1.0 | 10.8 | 12.2 | 20.5 | 55.6 |
| 65-69 | 0.0 | 0.6 | 5.7 | 8.7 | 85.0 |  | 0.0 | 2.2 | 7.6 | 7.6 | 82.6 |
| 70-74 | 0.0 | 0.0 | 0.1 | 2.7 | 97.2 |  | 0.0 | 0.0 | 2.2 | 6.0 | 91.8 |
| 75-79 | 0.0 | 0.0 | 0.0 | 0.0 | 100.0 |  | 0.0 | 0.0 | 0.0 | 0.9 | 99.1 |

Data are percentages. CKB: China Kadoorie Biobank; ASCVD: atherosclerotic cardiovascular disease.

# eTable 5. Age-sex-specific number of Chinese population free of ASCVD

| Age group (year) | Population number ^a^ | Prevalence of IHD (%)^b^ | Prevalence of IS (%)^b^ | Population number free of ASCVD |
| --- | --- | --- | --- | --- |
| Men |  |  |  |  |
| 30-34 | 60,621,795 | 0.36 | 0.24 | 60,257,664 |
| 35-39 | 50,328,205 | 0.69 | 0.33 | 49,816,504 |
| 40-44 | 50,300,000 | 1.27 | 0.45 | 49,434,417 |
| 45-49 | 62,374,359 | 2.17 | 0.69 | 60,592,035 |
| 50-54 | 60,314,103 | 3.48 | 1.12 | 57,545,408 |
| 55-59 | 47,670,513 | 5.23 | 1.94 | 44,249,852 |
| 60-64 | 39,061,538 | 7.49 | 3.23 | 34,873,872 |
| 65-69 | 34,573,077 | 10.69 | 5.05 | 29,128,938 |
| 70-74 | 22,167,949 | 14.07 | 7.17 | 17,458,590 |
| 75-79 | 13,548,718 | 18.57 | 9.31 | 9,772,143 |
| Women |  |  |  |  |
| 30-34 | 59,853,846 | 0.32 | 0.35 | 59,448,830 |
| 35-39 | 49,291,026 | 0.61 | 0.43 | 48,779,621 |
| 40-44 | 48,474,359 | 1.08 | 0.55 | 47,682,809 |
| 45-49 | 60,216,667 | 1.80 | 0.78 | 58,667,838 |
| 50-54 | 59,075,641 | 2.84 | 1.21 | 56,684,894 |
| 55-59 | 47,289,744 | 4.33 | 2.16 | 44,220,856 |
| 60-64 | 38,774,359 | 6.35 | 3.78 | 34,848,560 |
| 65-69 | 36,050,000 | 9.27 | 6.36 | 30,416,286 |
| 70-74 | 23,556,410 | 12.55 | 9.63 | 18,333,816 |
| 75-79 | 15,438,462 | 16.93 | 12.86 | 10,839,515 |

ASCVD: atherosclerotic cardiovascular disease; IHD: ischemic heart disease; IS: ischemic stroke.

^a^ Data from China Statistics Yearbook 2,020.

^b^ Data from the Global Burden of Disease Study 2,019.

# eTable 6. 10-year risk distribution of the study population with risk estimated by CKB-ASCVD soft outcome model

|  | All (n=28,624) | Men (n=11,158) | Women (n=17,466) |
| --- | --- | --- | --- |
| 10-year risk category at baseline | | | |
| Risk scores, % | 9.6 (4.4-20.1) | 11.2 (5.2-22.8) | 8.7 (3.9-18.3) |
| Risk score grouping |  |  |  |
| <5.0% | 8,214 (28.7) | 2,644 (23.7) | 5,570 (31.9) |
| 5.0-9.9% | 6,521 (22.8) | 2,458 (22.0) | 4,063 (23.3) |
| 10.0-14.9% | 3,986 (13.9) | 1,641 (14.7) | 2,345 (13.4) |
| 15.0-19.9% | 2,701 (9.4) | 1,113 (10.0) | 1,588 (9.1) |
| ≥20.0% | 7,202 (25.2) | 3,302 (29.6) | 3,900 (22.3) |
| 10-year risk category at first resurvey | | | |
| Risk scores, % ^a^ | 11.7 (5.6, 23.8) | 13.1 (6.3, 26.1) | 10.8 (5.2, 22.5) |
| Risk score grouping ^a^ |  |  |  |
| <5.0% | 4,066 (21.9) | 1,339 (18.5) | 2,727 (24.2) |
| 5.0-9.9% | 4,112 (22.2) | 1,545 (21.3) | 2,567 (22.7) |
| 10.0-14.9% | 2,776 (15.0) | 1,114 (15.4) | 1,662 (14.7) |
| 15.0-19.9% | 1,865 (10.1) | 790 (10.9) | 1,075 (9.5) |
| ≥20.0% | 5,712 (30.8) | 2,455 (33.9) | 3,257 (28.9) |
| 10-year risk category at second resurvey | | | |
| Risk scores, % ^a^ | 17.9 (9.1, 32.3) | 20.1 (10.6, 35.9) | 16.6 (8.4, 30.1) |
| Risk score grouping ^a^ |  |  |  |
| <5.0% | 2,343 (10.5) | 705 (8.3) | 1,638 (11.8) |
| 5.0-9.9% | 3,940 (17.6) | 1,273 (15.0) | 2,667 (19.2) |
| 10.0-14.9% | 3,259 (14.5) | 1,179 (13.9) | 2,080 (14.9) |
| 15.0-19.9% | 2,726 (12.2) | 1,065 (12.5) | 1,661 (11.9) |
| ≥20.0% | 10,152 (45.3) | 4,279 (50.3) | 5,873 (42.2) |

Data are numbers (percentages) or median (25–75th percentile range).

^a^ Risk scores were calculated and categorized in participants without ASCVD.

# eTable 7. Comparisons of all screening interval protocols with 3-yearly screening protocol with 10-year risk estimated by CKB-ASCVD hard outcome model

| Screen interval  strategy | Person-years spent unidentified in high-risk category  (million) | Difference in person-years spent unidentified in high-risk category  (million) | Treatment-related | | | |  | Health-check related | | | Difference in total cost (billion, CN¥) |
| --- | --- | --- | --- | --- | --- | --- | --- | --- | --- | --- | --- |
|  |  |  | Number of ASCVD events prevented  (thousand) | QALYs gained  (thousand) | Costs owing to statin treatment (billion, CN¥) | |  | Number of health-check times (million) | Costs owing to health check (billion, CN¥) | |  |
| 3-3 (Ref) | 251 (241, 262) | 0 (Ref) | 0 (Ref) | 0 (Ref) | 0 (Ref) | |  | 1,802 (1,778, 1,827) | 54.1 (53.3, 54.8) | | 0 (Ref) |
| 3-1 | 34 (33, 35) | -217 (-219, -216) | 544 (541, 547) | 490 (486, 492) | 46.2 (45.9, 46.4) | |  | 2,671 (2,617, 2,726) | 80.1 (78.5, 81.8) | | 72.2 (70.3, 74.1) |
| 3-2 | 148 (141, 155) | -103 (-110, -96) | 259 (242, 275) | 233 (217, 248) | 21.9 (20.5, 23.3) | |  | 2,015 (1,983, 2,048) | 60.5 (59.5, 61.4) | | 28.3 (25.9, 30.7) |
| 4-1 | 67 (65, 70) | -184 (-187, -182) | 461 (455, 467) | 415 (409, 420) | 39.1 (38.6, 39.6) | |  | 2,313 (2,266, 2,358) | 69.4 (68.0, 70.7) | | 54.4 (52.5, 56.3) |
| 4-2 | 169 (162, 176) | -82 (-89, -75) | 206 (188, 223) | 185 (169, 201) | 17.5 (16.0, 18.9) | |  | 1,727 (1,699, 1,754) | 51.8 (51.0, 52.6) | | 15.2 (12.9, 17.5) |
| 5-1 | 114 (110, 119) | -137 (-141, -133) | 343 (332, 353) | 309 (299, 317) | 29.1 (28.2, 29.9) | |  | 2,096 (2,056, 2,135) | 62.9 (61.7, 64.1) | | 37.9 (35.8, 39.9) |
| 5-2 | 210 (202, 218) | -42 (-50, -33) | 105 (84, 125) | 94 (75, 112) | 8.9 (7.1, 10.6) | |  | 1,547 (1,524, 1,569) | 46.4 (45.7, 47.1) | | 1.2 (-1.3, 3.6) |
| 6-1 | 158 (153, 164) | -93 (-98, -88) | 233 (219, 246) | 209 (197, 221) | 19.7 (18.6, 20.8) | |  | 1,879 (1,844, 1,914) | 56.4 (55.3, 57.4) | | 22.0 (19.8, 24.2) |
| 6-2 | 248 (239, 257) | -4 (-13, 6) | 9 (-14, 31) | 8 (-13, 28) | 0.8 (-1.2, 2.7) | |  | 1,366 (1,345, 1,385) | 41.0 (40.4, 41.6) | | -12.3 (-14.9, -9.9) |
| Risk cut-offs: 2.5% 5.0% | |  |  |  | |  |  |  | |  |  |
| 6-3-1 | 55 (52, 57) | -197 (-199, -194) | 493 (485, 499) | 443 (436, 448) | | 41.8 (41.2, 42.3) |  | 2,194 (2,140, 2,251) | | 65.8 (64.2, 67.5) | 53.5 (51.3, 55.7) |
| 7-3-1 | 68 (65, 71) | -183 (-186, -180) | 459 (450, 466) | 412 (405, 419) | | 38.9 (38.2, 39.5) |  | 2,177 (2,123, 2,233) | | 65.3 (63.7, 67.0) | 50.1 (47.8, 52.4) |
| 8-3-1 | 91 (87, 95) | -160 (-164, -156) | 402 (392, 410) | 361 (352, 369) | | 34.1 (33.2, 34.8) |  | 2,103 (2,052, 2,155) | | 63.1 (61.5, 64.7) | 43.1 (40.7, 45.4) |
| 6-3-2 | 157 (150, 165) | -94 (-101, -87) | 236 (217, 254) | 212 (195, 228) | | 20.0 (18.4, 21.5) |  | 1,604 (1,571, 1,639) | | 48.1 (47.1, 49.2) | 14.1 (11.5, 16.6) |
| 7-3-2 | 172 (165, 180) | -79 (-87, -71) | 198 (178, 217) | 178 (160, 195) | | 16.8 (15.1, 18.4) |  | 1,578 (1,545, 1,611) | | 47.3 (46.3, 48.3) | 10.1 (7.3, 12.7) |
| 8-3-2 | 191 (183, 199) | -61 (-69, -52) | 152 (131, 172) | 137 (118, 155) | | 12.9 (11.1, 14.6) |  | 1,529 (1,498, 1,561) | | 45.9 (44.9, 46.8) | 4.7 (2.0, 7.4) |
| 6-4-1 | 81 (77, 84) | -171 (-174, -167) | 427 (418, 436) | 384 (376, 392) | | 36.3 (35.4, 37.0) |  | 2,072 (2,023, 2,123) | | 62.2 (60.7, 63.7) | 44.3 (42.1, 46.6) |
| 7-4-1 | 95 (91, 99) | -157 (-160, -152) | 392 (381, 402) | 353 (343, 361) | | 33.2 (32.3, 34.1) |  | 2,020 (1,973, 2,068) | | 60.6 (59.2, 62.0) | 39.8 (37.5, 42.0) |
| 8-4-1 | 110 (106, 115) | -141 (-145, -137) | 353 (342, 364) | 318 (307, 327) | | 30.0 (29.0, 30.9) |  | 1,988 (1,942, 2,035) | | 59.6 (58.3, 61.0) | 35.5 (33.2, 37.8) |
| 6-4-2 | 177 (169, 185) | -75 (-82, -67) | 187 (167, 206) | 168 (150, 185) | | 15.9 (14.1, 17.5) |  | 1,520 (1,489, 1,552) | | 45.6 (44.7, 46.5) | 7.4 (4.7, 10.0) |
| 7-4-2 | 192 (184, 201) | -59 (-67, -50) | 148 (126, 168) | 133 (113, 151) | | 12.5 (10.7, 14.2) |  | 1,458 (1,429, 1,487) | | 43.7 (42.9, 44.6) | 2.2 (-0.5, 4.8) |
| 8-4-2 | 203 (195, 212) | -48 (-56, -39) | 120 (98, 140) | 108 (89, 126) | | 10.2 (8.4, 11.9) |  | 1,452 (1,423, 1,481) | | 43.6 (42.7, 44.4) | -0.3 (-3.0, 2.3) |
| 6-5-1 | 119 (114, 124) | -132 (-137, -127) | 332 (318, 343) | 298 (286, 309) | | 28.1 (27.0, 29.1) |  | 1,940 (1,897, 1,985) | | 58.2 (56.9, 59.5) | 32.3 (29.8, 34.6) |
| 7-5-1 | 122 (117, 127) | -130 (-134, -124) | 324 (311, 336) | 292 (280, 302) | | 27.5 (26.4, 28.5) |  | 1,923 (1,881, 1,967) | | 57.7 (56.4, 59.0) | 31.1 (28.8, 33.4) |
| 8-5-1 | 137 (132, 143) | -114 (-119, -109) | 286 (272, 298) | 257 (245, 268) | | 24.2 (23.1, 25.3) |  | 1,891 (1,850, 1,934) | | 56.7 (55.5, 58.0) | 26.9 (24.5, 29.2) |
| 6-5-2 | 212 (203, 221) | -40 (-48, -30) | 99 (75, 121) | 89 (68, 109) | | 8.4 (6.4, 10.2) |  | 1,405 (1,379, 1,432) | | 42.1 (41.4, 42.9) | -3.5 (-6.3, -0.9) |
| 7-5-2 | 216 (208, 226) | -35 (-44, -26) | 88 (64, 109) | 79 (58, 98) | | 7.4 (5.5, 9.3) |  | 1,378 (1,354, 1,404) | | 41.4 (40.6, 42.1) | -5.3 (-8.0, -2.7) |
| 8-5-2 | 227 (219, 237) | -24 (-33, -15) | 60 (37, 82) | 54 (33, 74) | | 5.1 (3.1, 7.0) |  | 1,372 (1,347, 1,398) | | 41.2 (40.4, 41.9) | -7.8 (-10.5, -5.2) |
| Risk cut-offs: 2.5% 7.5% | |  |  |  |  | |  |  |  | |  |
| 6-3-1 | 89 (85, 95) | -162 (-166, -156) | 407 (392, 417) | 366 (352, 375) | 34.5 (33.2, 35.3) | |  | 1,697 (1,653, 1,740) | 50.9 (49.6, 52.2) | | 31.3 (28.7, 33.5) |
| 7-3-1 | 104 (100, 110) | -148 (-152, -141) | 369 (354, 380) | 332 (318, 342) | 31.3 (30.0, 32.2) | |  | 1,688 (1,645, 1,731) | 50.6 (49.3, 51.9) | | 27.9 (25.3, 30.1) |
| 8-3-1 | 129 (124, 136) | -122 (-127, -115) | 306 (288, 318) | 275 (259, 286) | 25.9 (24.4, 27.0) | |  | 1,603 (1,563, 1,644) | 48.1 (46.9, 49.3) | | 20.0 (17.2, 22.2) |
| 6-3-2 | 170 (161, 181) | -81 (-90, -70) | 203 (176, 226) | 183 (158, 203) | 17.2 (14.9, 19.1) | |  | 1,488 (1,458, 1,516) | 44.6 (43.8, 45.5) | | 7.8 (4.6, 10.5) |
| 7-3-2 | 186 (177, 197) | -65 (-74, -54) | 163 (135, 186) | 147 (121, 168) | 13.8 (11.4, 15.8) | |  | 1,476 (1,447, 1,504) | 44.3 (43.4, 45.1) | | 4.0 (0.8, 6.9) |
| 8-3-2 | 209 (199, 221) | -42 (-52, -30) | 106 (76, 130) | 95 (69, 117) | 9.0 (6.5, 11.0) | |  | 1,399 (1,372, 1,425) | 42.0 (41.2, 42.8) | | -3.1 (-6.4, -0.3) |
| 6-4-1 | 139 (133, 147) | -113 (-118, -105) | 282 (262, 296) | 253 (235, 266) | 23.9 (22.2, 25.1) | |  | 1,512 (1,476, 1,549) | 45.4 (44.3, 46.5) | | 15.2 (12.4, 17.5) |
| 7-4-1 | 155 (149, 164) | -96 (-102, -88) | 241 (219, 256) | 216 (197, 230) | 20.4 (18.6, 21.7) | |  | 1,442 (1,409, 1,477) | 43.3 (42.3, 44.3) | | 9.6 (6.8, 12.0) |
| 8-4-1 | 169 (163, 178) | -82 (-88, -73) | 205 (184, 221) | 185 (165, 199) | 17.4 (15.6, 18.7) | |  | 1,426 (1,393, 1,460) | 42.8 (41.8, 43.8) | | 6.1 (3.3, 8.5) |
| 6-4-2 | 210 (200, 222) | -42 (-51, -29) | 104 (74, 129) | 94 (66, 116) | 8.8 (6.3, 10.9) | |  | 1,330 (1,305, 1,355) | 39.9 (39.2, 40.6) | | -5.3 (-8.6, -2.5) |
| 7-4-2 | 227 (217, 240) | -24 (-34, -11) | 60 (28, 86) | 54 (26, 77) | 5.1 (2.4, 7.3) | |  | 1,258 (1,236, 1,280) | 37.7 (37.1, 38.4) | | -11.2 (-14.6, -8.4) |
| 8-4-2 | 239 (229, 251) | -13 (-23, 0) | 31 (0, 57) | 28 (0, 51) | 2.7 (0.0, 4.8) | |  | 1,249 (1,226, 1,271) | 37.5 (36.8, 38.1) | | -13.9 (-17.3, -11.1) |
| 6-5-1 | 208 (200, 219) | -43 (-51, -32) | 108 (80, 128) | 97 (72, 115) | 9.1 (6.8, 10.9) | |  | 1,354 (1,323, 1,385) | 40.6 (39.7, 41.6) | | -4.3 (-7.6, -1.7) |
| 7-5-1 | 208 (201, 219) | -43 (-51, -32) | 107 (81, 127) | 96 (73, 114) | 9.1 (6.9, 10.7) | |  | 1,343 (1,312, 1,374) | 40.3 (39.4, 41.2) | | -4.7 (-7.8, -2.1) |
| 8-5-1 | 223 (215, 233) | -29 (-37, -18) | 72 (45, 91) | 65 (41, 82) | 6.1 (3.8, 7.8) | |  | 1,327 (1,296, 1,357) | 39.8 (38.9, 40.7) | | -8.2 (-11.3, -5.6) |
| 6-5-2 | 271 (260, 286) | 20 (8, 35) | -50 (-86, -21) | -45 (-78, -19) | -4.3 (-7.3, -1.8) | |  | 1,191 (1,170, 1,211) | 35.7 (35.1, 36.3) | | -22.6 (-26.3, -19.5) |
| 7-5-2 | 273 (261, 287) | 21 (10, 35) | -54 (-89, -25) | -48 (-80, -22) | -4.5 (-7.5, -2.1) | |  | 1,177 (1,157, 1,196) | 35.3 (34.7, 35.9) | | -23.3 (-26.9, -20.3) |
| 8-5-2 | 284 (273, 298) | 33 (22, 47) | -82 (-117, -54) | -74 (-105, -48) | -7.0 (-9.9, -4.6) | |  | 1,168 (1,147, 1,187) | 35.0 (34.4, 35.6) | | -26.0 (-29.6, -23.0) |
| Risk cut-offs: 5.0% 7.5% | |  |  |  |  | |  |  |  | |  |
| 6-3-1 | 134 (128, 141) | -117 (-123, -110) | 294 (276, 308) | 264 (248, 277) | 24.9 (23.4, 26.1) | |  | 1,388 (1,349, 1,430) | 41.6 (40.5, 42.9) | | 12.5 (9.8, 15.0) |
| 7-3-1 | 166 (160, 174) | -85 (-91, -77) | 213 (193, 229) | 192 (174, 206) | 18.1 (16.4, 19.4) | |  | 1,362 (1,324, 1,403) | 40.9 (39.7, 42.1) | | 4.9 (2.1, 7.4) |
| 8-3-1 | 220 (213, 230) | -31 (-38, -21) | 77 (54, 96) | 70 (48, 87) | 6.6 (4.5, 8.2) | |  | 1,297 (1,262, 1,334) | 38.9 (37.9, 40.0) | | -8.6 (-11.7, -5.9) |
| 6-3-2 | 206 (196, 218) | -45 (-55, -33) | 113 (83, 138) | 101 (75, 124) | 9.6 (7.1, 11.7) | |  | 1,210 (1,185, 1,235) | 36.3 (35.6, 37.1) | | -8.2 (-11.4, -5.3) |
| 7-3-2 | 240 (229, 252) | -11 (-22, 1) | 29 (-2, 56) | 26 (-2, 50) | 2.4 (-0.2, 4.8) | |  | 1,181 (1,157, 1,205) | 35.4 (34.7, 36.1) | | -16.2 (-19.6, -13.2) |
| 8-3-2 | 288 (277, 302) | 37 (25, 50) | -93 (-126, -63) | -83 (-114, -57) | -7.9 (-10.7, -5.4) | |  | 1,131 (1,109, 1,153) | 33.9 (33.3, 34.6) | | -28.0 (-31.5, -24.8) |
| 6-4-1 | 175 (168, 184) | -76 (-83, -67) | 190 (168, 208) | 171 (151, 187) | 16.1 (14.2, 17.7) | |  | 1,329 (1,294, 1,366) | 39.9 (38.8, 41.0) | | 1.9 (-1.1, 4.6) |
| 7-4-1 | 209 (201, 218) | -43 (-51, -33) | 106 (82, 127) | 96 (74, 114) | 9.0 (7.0, 10.7) | |  | 1,278 (1,246, 1,313) | 38.4 (37.4, 39.4) | | -6.7 (-9.7, -3.9) |
| 8-4-1 | 245 (237, 256) | -6 (-14, 4) | 15 (-11, 36) | 13 (-10, 32) | 1.2 (-0.9, 3.0) | |  | 1,244 (1,213, 1,277) | 37.3 (36.4, 38.3) | | -15.5 (-18.6, -12.7) |
| 6-4-2 | 242 (231, 255) | -9 (-20, 4) | 22 (-10, 50) | 20 (-9, 45) | 1.9 (-0.9, 4.3) | |  | 1,164 (1,142, 1,187) | 34.9 (34.3, 35.6) | | -17.3 (-20.7, -14.2) |
| 7-4-2 | 277 (265, 291) | 26 (14, 40) | -65 (-99, -34) | -58 (-89, -31) | -5.5 (-8.4, -2.9) | |  | 1,110 (1,091, 1,130) | 33.3 (32.7, 33.9) | | -26.2 (-29.7, -23.1) |
| 8-4-2 | 308 (296, 322) | 57 (45, 70) | -142 (-176, -112) | -128 (-159, -101) | -12.0 (-15.0, -9.5) | |  | 1,092 (1,072, 1,111) | 32.7 (32.2, 33.3) | | -33.4 (-36.9, -30.2) |
| 6-5-1 | 232 (223, 243) | -19 (-28, -8) | 48 (20, 71) | 43 (18, 64) | 4.0 (1.7, 6.1) | |  | 1,266 (1,235, 1,300) | 38.0 (37.1, 39.0) | | -12.0 (-15.3, -9.0) |
| 7-5-1 | 241 (232, 252) | -10 (-19, 0) | 26 (-1, 48) | 23 (-1, 43) | 2.2 (-0.1, 4.1) | |  | 1,241 (1,211, 1,273) | 37.2 (36.3, 38.2) | | -14.7 (-17.8, -11.8) |
| 8-5-1 | 278 (268, 289) | 26 (17, 38) | -66 (-94, -43) | -60 (-85, -39) | -5.6 (-8.0, -3.6) | |  | 1,207 (1,178, 1,238) | 36.2 (35.3, 37.1) | | -23.5 (-26.7, -20.6) |
| 6-5-2 | 296 (282, 310) | 44 (31, 59) | -111 (-148, -78) | -100 (-133, -70) | -9.4 (-12.6, -6.6) | |  | 1,110 (1,092, 1,129) | 33.3 (32.7, 33.9) | | -30.2 (-33.9, -26.8) |
| 7-5-2 | 306 (293, 320) | 54 (42, 69) | -136 (-172, -104) | -123 (-155, -94) | -11.6 (-14.6, -8.8) | |  | 1,082 (1,064, 1,100) | 32.4 (31.9, 33.0) | | -33.2 (-36.8, -29.9) |
| 8-5-2 | 337 (324, 351) | 85 (73, 100) | -214 (-250, -182) | -192 (-225, -164) | -18.1 (-21.2, -15.4) | |  | 1,063 (1,045, 1,081) | 31.9 (31.3, 32.4) | | -40.3 (-43.9, -37.1) |

CKB: China Kadoorie Biobank; ASCVD: atherosclerotic cardiovascular disease; QALY: quality-adjusted life year; CN¥: Chinese Yuan.

Data are estimated with 95% CIs over a 10-year period.

# eTable 8. Comparisons of all screening interval protocols with 3-yearly screening protocol with 10-year risk estimated by CKB-ASCVD soft outcome model

| Screen interval  strategy | Person-years spent unidentified in high-risk category  (million) | Difference in person-years spent unidentified in high-risk category  (million) | Treatment-related | | | |  | Health-check related | | | Difference in total cost (billion, CN¥) |
| --- | --- | --- | --- | --- | --- | --- | --- | --- | --- | --- | --- |
|  |  |  | Number of ASCVD events prevented  (thousand) | QALYs gained  (thousand) | Costs owing to statin treatment (billion, CN¥) | |  | Number of health-check times (million) | Costs owing to health check (billion, CN¥) | |  |
| 3-3 (Ref) | 245 (236, 255) | 0 (Ref) | 0 (Ref) | 0 (Ref) | 0 (Ref) | |  | 1,945 (1,919, 1,970) | 58.3 (57.6, 59.1) | | 0 (Ref) |
| 3-1 | 30 (28, 31) | -216 (-217, -215) | 540 (537, 543) | 486 (483, 488) | 45.8 (45.6, 46.1) | |  | 2,957 (2,902, 3,011) | 88.7 (87.1, 90.3) | | 76.2 (74.3, 78.1) |
| 3-2 | 143 (137, 150) | -102 (-108, -96) | 256 (240, 271) | 230 (216, 244) | 21.7 (20.3, 23.0) | |  | 2,195 (2,161, 2,228) | 65.9 (64.8, 66.8) | | 29.2 (26.8, 31.5) |
| 4-1 | 59 (57, 61) | -187 (-189, -184) | 468 (462, 473) | 420 (415, 425) | 39.6 (39.1, 40.1) | |  | 2,581 (2,535, 2,626) | 77.4 (76.0, 78.8) | | 58.7 (56.8, 60.5) |
| 4-2 | 161 (155, 168) | -84 (-91, -78) | 211 (194, 227) | 190 (175, 204) | 17.9 (16.5, 19.2) | |  | 1,895 (1,866, 1,923) | 56.8 (56.0, 57.7) | | 16.4 (14.1, 18.6) |
| 5-1 | 100 (97, 104) | -145 (-148, -141) | 363 (354, 371) | 326 (318, 334) | 30.8 (30.0, 31.5) | |  | 2,350 (2,309, 2,388) | 70.5 (69.3, 71.6) | | 42.9 (40.9, 44.8) |
| 5-2 | 197 (190, 205) | -48 (-56, -41) | 121 (102, 139) | 109 (92, 125) | 10.3 (8.6, 11.8) | |  | 1,701 (1,676, 1,723) | 51.0 (50.3, 51.7) | | 2.9 (0.6, 5.2) |
| 6-1 | 139 (135, 144) | -106 (-110, -101) | 265 (253, 277) | 239 (228, 249) | 22.5 (21.5, 23.5) | |  | 2,121 (2,087, 2,155) | 63.6 (62.6, 64.6) | | 27.8 (25.7, 29.8) |
| 6-2 | 230 (222, 239) | -15 (-23, -7) | 38 (17, 58) | 34 (15, 52) | 3.2 (1.4, 4.9) | |  | 1,512 (1,492, 1,532) | 45.4 (44.8, 46.0) | | -9.8 (-12.2, -7.5) |
| Risk cut-offs: 5.0% 10.0% | |  |  |  | |  |  |  | |  |  |
| 6-3-1 | 46 (44, 49) | -199 (-201, -197) | 498 (492, 503) | 448 (443, 453) | | 42.3 (41.7, 42.7) |  | 2,493 (2,439, 2,549) | | 74.8 (73.2, 76.5) | 58.7 (56.6, 60.8) |
| 7-3-1 | 58 (56, 61) | -187 (-190, -185) | 469 (462, 475) | 422 (416, 427) | | 39.8 (39.2, 40.3) |  | 2,477 (2,423, 2,532) | | 74.3 (72.7, 76.0) | 55.8 (53.6, 57.9) |
| 8-3-1 | 77 (74, 81) | -168 (-171, -165) | 421 (412, 428) | 378 (371, 385) | | 35.7 (35.0, 36.3) |  | 2,395 (2,343, 2,447) | | 71.8 (70.3, 73.4) | 49.2 (46.9, 51.4) |
| 6-3-2 | 150 (143, 157) | -96 (-102, -89) | 239 (222, 256) | 215 (199, 230) | | 20.3 (18.8, 21.7) |  | 1,793 (1,759, 1,829) | | 53.8 (52.8, 54.9) | 15.8 (13.2, 18.2) |
| 7-3-2 | 163 (156, 170) | -82 (-89, -75) | 206 (188, 224) | 186 (169, 201) | | 17.5 (15.9, 19.0) |  | 1,766 (1,733, 1,800) | | 53.0 (52.0, 54.0) | 12.1 (9.6, 14.6) |
| 8-3-2 | 178 (171, 186) | -67 (-74, -59) | 168 (149, 186) | 151 (134, 167) | | 14.3 (12.6, 15.8) |  | 1,712 (1,680, 1,745) | | 51.4 (50.4, 52.3) | 7.3 (4.7, 9.8) |
| 6-4-1 | 70 (67, 73) | -176 (-179, -172) | 440 (432, 447) | 396 (388, 402) | | 37.3 (36.6, 37.9) |  | 2,353 (2,304, 2,403) | | 70.6 (69.1, 72.1) | 49.5 (47.4, 51.7) |
| 7-4-1 | 82 (78, 85) | -164 (-167, -160) | 410 (400, 418) | 368 (360, 376) | | 34.7 (34.0, 35.4) |  | 2,294 (2,247, 2,342) | | 68.8 (67.4, 70.3) | 45.2 (43.0, 47.4) |
| 8-4-1 | 95 (91, 99) | -151 (-154, -147) | 377 (367, 386) | 339 (330, 347) | | 32.0 (31.2, 32.8) |  | 2,262 (2,216, 2,308) | | 67.9 (66.5, 69.2) | 41.5 (39.3, 43.7) |
| 6-4-2 | 167 (160, 174) | -79 (-86, -71) | 197 (178, 214) | 177 (160, 193) | | 16.7 (15.1, 18.2) |  | 1,696 (1,665, 1,728) | | 50.9 (50.0, 51.8) | 9.2 (6.7, 11.7) |
| 7-4-2 | 180 (173, 188) | -65 (-72, -57) | 163 (143, 181) | 146 (128, 163) | | 13.8 (12.1, 15.4) |  | 1,627 (1,598, 1,656) | | 48.8 (47.9, 49.7) | 4.2 (1.7, 6.7) |
| 8-4-2 | 189 (182, 197) | -56 (-64, -48) | 141 (121, 159) | 126 (109, 143) | | 11.9 (10.2, 13.5) |  | 1,622 (1,594, 1,652) | | 48.7 (47.8, 49.6) | 2.3 (-0.3, 4.7) |
| 6-5-1 | 104 (100, 109) | -141 (-145, -136) | 353 (341, 363) | 317 (307, 327) | | 29.9 (28.9, 30.8) |  | 2,198 (2,155, 2,242) | | 65.9 (64.7, 67.3) | 37.5 (35.3, 39.7) |
| 7-5-1 | 106 (102, 111) | -139 (-143, -134) | 348 (336, 358) | 313 (303, 322) | | 29.5 (28.5, 30.3) |  | 2,182 (2,139, 2,225) | | 65.5 (64.2, 66.7) | 36.6 (34.4, 38.7) |
| 8-5-1 | 119 (115, 124) | -126 (-130, -121) | 315 (303, 326) | 284 (273, 293) | | 26.7 (25.7, 27.7) |  | 2,149 (2,108, 2,191) | | 64.5 (63.2, 65.7) | 32.9 (30.6, 35.0) |
| 6-5-2 | 199 (191, 207) | -47 (-55, -38) | 117 (95, 137) | 105 (86, 123) | | 9.9 (8.1, 11.6) |  | 1,560 (1,534, 1,587) | | 46.8 (46.0, 47.6) | -1.6 (-4.2, 0.9) |
| 7-5-2 | 202 (194, 211) | -43 (-51, -34) | 108 (86, 127) | 97 (78, 114) | | 9.1 (7.3, 10.8) |  | 1,533 (1,508, 1,558) | | 46.0 (45.2, 46.7) | -3.2 (-5.8, -0.8) |
| 8-5-2 | 211 (203, 220) | -34 (-42, -26) | 86 (64, 105) | 77 (58, 95) | | 7.3 (5.4, 8.9) |  | 1,528 (1,503, 1,554) | | 45.9 (45.1, 46.6) | -5.2 (-7.8, -2.8) |
| Risk cut-offs: 5.0% 15.0% | |  |  |  |  | |  |  |  | |  |
| 6-3-1 | 78 (75, 82) | -167 (-171, -163) | 419 (409, 428) | 377 (368, 385) | 35.6 (34.7, 36.3) | |  | 1,891 (1,848, 1,934) | 56.7 (55.4, 58.0) | | 33.9 (31.8, 35.9) |
| 7-3-1 | 91 (87, 95) | -154 (-158, -150) | 386 (375, 396) | 347 (338, 356) | 32.8 (31.8, 33.5) | |  | 1,884 (1,841, 1,926) | 56.5 (55.2, 57.8) | | 30.9 (28.7, 33.0) |
| 8-3-1 | 113 (109, 118) | -132 (-136, -127) | 331 (318, 341) | 297 (286, 307) | 28.0 (27.0, 28.9) | |  | 1,790 (1,750, 1,830) | 53.7 (52.5, 54.9) | | 23.4 (21.1, 25.5) |
| 6-3-2 | 163 (154, 172) | -83 (-91, -74) | 207 (185, 228) | 186 (166, 205) | 17.6 (15.7, 19.3) | |  | 1,646 (1,617, 1,675) | 49.4 (48.5, 50.2) | | 8.6 (5.8, 11.2) |
| 7-3-2 | 177 (168, 186) | -68 (-77, -59) | 171 (148, 193) | 154 (133, 173) | 14.5 (12.5, 16.3) | |  | 1,635 (1,607, 1,663) | 49.1 (48.2, 49.9) | | 5.2 (2.4, 7.9) |
| 8-3-2 | 196 (188, 206) | -49 (-58, -39) | 122 (98, 145) | 110 (88, 130) | 10.4 (8.3, 12.3) | |  | 1,549 (1,523, 1,576) | 46.5 (45.7, 47.3) | | -1.5 (-4.4, 1.2) |
| 6-4-1 | 124 (119, 130) | -122 (-127, -116) | 304 (290, 317) | 274 (261, 285) | 25.8 (24.6, 26.9) | |  | 1,676 (1,640, 1,713) | 50.3 (49.2, 51.4) | | 17.8 (15.4, 19.9) |
| 7-4-1 | 138 (133, 144) | -107 (-112, -101) | 268 (253, 282) | 241 (227, 253) | 22.7 (21.4, 23.9) | |  | 1,598 (1,565, 1,633) | 48.0 (46.9, 49.0) | | 12.3 (10.0, 14.5) |
| 8-4-1 | 151 (145, 157) | -95 (-100, -88) | 237 (221, 251) | 213 (199, 226) | 20.1 (18.8, 21.3) | |  | 1,582 (1,549, 1,616) | 47.5 (46.5, 48.5) | | 9.3 (6.9, 11.4) |
| 6-4-2 | 198 (189, 208) | -47 (-56, -37) | 118 (94, 141) | 107 (84, 127) | 10.0 (8.0, 12.0) | |  | 1,462 (1,437, 1,486) | 43.9 (43.1, 44.6) | | -4.4 (-7.3, -1.8) |
| 7-4-2 | 214 (204, 224) | -32 (-41, -21) | 79 (53, 103) | 71 (48, 93) | 6.7 (4.5, 8.7) | |  | 1,381 (1,359, 1,403) | 41.4 (40.8, 42.1) | | -10.2 (-13.1, -7.5) |
| 8-4-2 | 223 (214, 234) | -22 (-31, -12) | 55 (30, 79) | 50 (27, 71) | 4.7 (2.5, 6.7) | |  | 1,373 (1,350, 1,395) | 41.2 (40.5, 41.8) | | -12.5 (-15.3, -9.8) |
| 6-5-1 | 188 (181, 196) | -57 (-64, -49) | 143 (122, 161) | 128 (110, 144) | 12.1 (10.4, 13.6) | |  | 1,494 (1,463, 1,526) | 44.8 (43.9, 45.8) | | -1.4 (-4.1, 1.1) |
| 7-5-1 | 188 (181, 196) | -57 (-64, -49) | 143 (123, 160) | 128 (111, 144) | 12.1 (10.4, 13.6) | |  | 1,484 (1,453, 1,515) | 44.5 (43.6, 45.5) | | -1.7 (-4.3, 0.7) |
| 8-5-1 | 201 (194, 209) | -45 (-52, -37) | 112 (92, 129) | 101 (83, 116) | 9.5 (7.8, 11.0) | |  | 1,468 (1,437, 1,499) | 44.0 (43.1, 45.0) | | -4.8 (-7.4, -2.4) |
| 6-5-2 | 255 (244, 267) | 10 (-1, 21) | -24 (-53, 3) | -22 (-48, 3) | -2.0 (-4.5, 0.3) | |  | 1,301 (1,280, 1,321) | 39.0 (38.4, 39.6) | | -21.4 (-24.4, -18.4) |
| 7-5-2 | 256 (245, 268) | 11 (0, 22) | -27 (-56, 0) | -24 (-50, 0) | -2.3 (-4.7, 0.0) | |  | 1,288 (1,267, 1,308) | 38.6 (38.0, 39.2) | | -22.0 (-25.0, -19.1) |
| 8-5-2 | 266 (255, 277) | 20 (10, 32) | -51 (-79, -25) | -46 (-71, -22) | -4.3 (-6.7, -2.1) | |  | 1,280 (1,259, 1,300) | 38.4 (37.8, 39.0) | | -24.3 (-27.3, -21.4) |
| Risk cut-offs: 10.0% 15.0% | |  |  |  |  | |  |  |  | |  |
| 6-3-1 | 111 (106, 116) | -135 (-139, -129) | 337 (323, 349) | 303 (291, 314) | 28.6 (27.4, 29.6) | |  | 1,548 (1,509, 1,589) | 46.4 (45.3, 47.7) | | 16.7 (14.4, 18.9) |
| 7-3-1 | 138 (132, 144) | -108 (-113, -102) | 269 (254, 283) | 242 (229, 254) | 22.8 (21.6, 24.0) | |  | 1,525 (1,487, 1,565) | 45.7 (44.6, 46.9) | | 10.2 (7.8, 12.6) |
| 8-3-1 | 184 (178, 192) | -61 (-68, -54) | 153 (135, 170) | 138 (121, 153) | 13.0 (11.4, 14.4) | |  | 1,451 (1,416, 1,488) | 43.5 (42.5, 44.6) | | -1.8 (-4.4, 0.7) |
| 6-3-2 | 187 (178, 197) | -58 (-67, -48) | 146 (121, 169) | 131 (109, 152) | 12.4 (10.3, 14.3) | |  | 1,332 (1,306, 1,357) | 40.0 (39.2, 40.7) | | -6.0 (-8.9, -3.3) |
| 7-3-2 | 216 (206, 226) | -29 (-39, -19) | 74 (47, 98) | 66 (42, 89) | 6.2 (4.0, 8.4) | |  | 1,303 (1,279, 1,328) | 39.1 (38.4, 39.8) | | -13.0 (-16.0, -10.2) |
| 8-3-2 | 256 (246, 268) | 11 (0, 22) | -27 (-56, -1) | -25 (-51, -1) | -2.3 (-4.8, -0.1) | |  | 1,247 (1,225, 1,270) | 37.4 (36.7, 38.1) | | -23.3 (-26.4, -20.3) |
| 6-4-1 | 149 (143, 156) | -96 (-102, -89) | 241 (223, 256) | 216 (201, 230) | 20.4 (18.9, 21.7) | |  | 1,477 (1,442, 1,513) | 44.3 (43.3, 45.4) | | 6.4 (3.8, 8.8) |
| 7-4-1 | 178 (171, 185) | -68 (-75, -60) | 170 (151, 187) | 153 (135, 168) | 14.4 (12.8, 15.9) | |  | 1,422 (1,390, 1,456) | 42.7 (41.7, 43.7) | | -1.3 (-3.9, 1.2) |
| 8-4-1 | 208 (200, 216) | -38 (-45, -29) | 94 (74, 112) | 85 (66, 101) | 8.0 (6.2, 9.5) | |  | 1,388 (1,357, 1,420) | 41.6 (40.7, 42.6) | | -8.7 (-11.4, -6.2) |
| 6-4-2 | 220 (210, 231) | -25 (-36, -15) | 64 (37, 89) | 57 (33, 80) | 5.4 (3.1, 7.6) | |  | 1,277 (1,254, 1,300) | 38.3 (37.6, 39.0) | | -14.6 (-17.6, -11.8) |
| 7-4-2 | 250 (239, 262) | 5 (-6, 16) | -12 (-41, 16) | -10 (-37, 14) | -1.0 (-3.5, 1.4) | |  | 1,218 (1,198, 1,237) | 36.5 (35.9, 37.1) | | -22.8 (-25.9, -19.9) |
| 8-4-2 | 274 (263, 286) | 29 (18, 41) | -72 (-102, -45) | -65 (-91, -41) | -6.1 (-8.6, -3.8) | |  | 1,201 (1,180, 1,221) | 36.0 (35.4, 36.6) | | -28.5 (-31.6, -25.6) |
| 6-5-1 | 203 (195, 212) | -42 (-50, -33) | 105 (82, 126) | 94 (74, 113) | 8.9 (7.0, 10.7) | |  | 1,398 (1,367, 1,429) | 41.9 (41.0, 42.9) | | -7.5 (-10.3, -4.8) |
| 7-5-1 | 209 (201, 217) | -37 (-44, -28) | 91 (70, 111) | 82 (63, 100) | 7.8 (5.9, 9.4) | |  | 1,374 (1,345, 1,405) | 41.2 (40.4, 42.2) | | -9.4 (-12.1, -6.8) |
| 8-5-1 | 239 (231, 248) | -6 (-15, 3) | 16 (-7, 36) | 14 (-6, 33) | 1.3 (-0.6, 3.1) | |  | 1,340 (1,312, 1,370) | 40.2 (39.3, 41.1) | | -16.8 (-19.6, -14.2) |
| 6-5-2 | 270 (258, 283) | 25 (13, 38) | -62 (-94, -32) | -56 (-84, -29) | -5.3 (-8.0, -2.7) | |  | 1,208 (1,189, 1,227) | 36.3 (35.7, 36.8) | | -27.4 (-30.6, -24.2) |
| 7-5-2 | 277 (266, 290) | 32 (20, 44) | -80 (-111, -51) | -72 (-100, -46) | -6.8 (-9.4, -4.3) | |  | 1,180 (1,162, 1,198) | 35.4 (34.9, 35.9) | | -29.7 (-32.9, -26.7) |
| 8-5-2 | 302 (290, 314) | 56 (45, 69) | -141 (-172, -112) | -127 (-155, -101) | -12.0 (-14.6, -9.5) | |  | 1,163 (1,145, 1,181) | 34.9 (34.3, 35.4) | | -35.4 (-38.6, -32.4) |

CKB: China Kadoorie Biobank; ASCVD: atherosclerotic cardiovascular disease; QALY: quality-adjusted life year; CN¥: Chinese Yuan.

Data are estimated with 95% CIs over a 10-year period.

# eTable 9. Comparisons of all screening interval protocols with 3-yearly screening protocol using the low limit of incremental costs of statin treatment

| Screen interval  strategy | Person-years spent unidentified in high-risk category  (million) | Difference in person-years spent unidentified in high-risk category  (million) | Treatment-related | | | |  | Health-check related | | | Difference in total cost (billion, CN¥) |
| --- | --- | --- | --- | --- | --- | --- | --- | --- | --- | --- | --- |
|  |  |  | Number of ASCVD events prevented  (thousand) | QALYs gained  (thousand) | Costs owing to statin treatment (billion, CN¥) | |  | Number of health-check times (million) | Costs owing to health check (billion, CN¥) | |  |
| 3-3 (Ref) | 251 (241, 262) | 0 (Ref) | 0 (Ref) | 0 (Ref) | 0 (Ref) | |  | 1,802 (1,778, 1,827) | 54.1 (53.3, 54.8) | | 0 (Ref) |
| 3-1 | 34 (33, 35) | -217 (-219, -216) | 544 (541, 547) | 490 (486, 492) | 15.4 (15.3, 15.5) | |  | 2,671 (2,617, 2,726) | 80.1 (78.5, 81.8) | | 41.5 (39.7, 43.2) |
| 3-2 | 148 (141, 155) | -103 (-110, -96) | 259 (242, 275) | 233 (217, 248) | 7.3 (6.8, 7.8) | |  | 2,015 (1,983, 2,048) | 60.5 (59.5, 61.4) | | 13.7 (12.2, 15.1) |
| 4-1 | 67 (65, 70) | -184 (-187, -182) | 461 (455, 467) | 415 (409, 420) | 13.0 (12.9, 13.2) | |  | 2,313 (2,266, 2,358) | 69.4 (68.0, 70.7) | | 28.4 (26.8, 29.9) |
| 4-2 | 169 (162, 176) | -82 (-89, -75) | 206 (188, 223) | 185 (169, 201) | 5.8 (5.3, 6.3) | |  | 1,727 (1,699, 1,754) | 51.8 (51.0, 52.6) | | 3.6 (2.2, 4.9) |
| 5-1 | 114 (110, 119) | -137 (-141, -133) | 343 (332, 353) | 309 (299, 317) | 9.7 (9.4, 10.0) | |  | 2,096 (2,056, 2,135) | 62.9 (61.7, 64.1) | | 18.5 (17.0, 19.9) |
| 5-2 | 210 (202, 218) | -42 (-50, -33) | 105 (84, 125) | 94 (75, 112) | 3.0 (2.4, 3.5) | |  | 1,547 (1,524, 1,569) | 46.4 (45.7, 47.1) | | -4.7 (-6.0, -3.5) |
| 6-1 | 158 (153, 164) | -93 (-98, -88) | 233 (219, 246) | 209 (197, 221) | 6.6 (6.2, 6.9) | |  | 1,879 (1,844, 1,914) | 56.4 (55.3, 57.4) | | 8.9 (7.4, 10.3) |
| 6-2 | 248 (239, 257) | -4 (-13, 6) | 9 (-14, 31) | 8 (-13, 28) | 0.3 (-0.4, 0.9) | |  | 1,366 (1,345, 1,385) | 41.0 (40.4, 41.6) | | -12.8 (-14.1, -11.6) |
| Risk cut-offs: 2.5% 5.0% | |  |  |  |  | |  |  |  | |  |
| 6-3-1 | 55 (52, 57) | -197 (-199, -194) | 493 (485, 499) | 443 (436, 448) | | 13.9 (13.7, 14.1) |  | 2,194 (2,140, 2,251) | | 65.8 (64.2, 67.5) | 25.7 (23.8, 27.6) |
| 7-3-1 | 68 (65, 71) | -183 (-186, -180) | 459 (450, 466) | 412 (405, 419) | | 13.0 (12.7, 13.2) |  | 2,177 (2,123, 2,233) | | 65.3 (63.7, 67.0) | 24.2 (22.3, 26.1) |
| 8-3-1 | 91 (87, 95) | -160 (-164, -156) | 402 (392, 410) | 361 (352, 369) | | 11.4 (11.1, 11.6) |  | 2,103 (2,052, 2,155) | | 63.1 (61.5, 64.7) | 20.4 (18.5, 22.2) |
| 6-3-2 | 157 (150, 165) | -94 (-101, -87) | 236 (217, 254) | 212 (195, 228) | | 6.7 (6.1, 7.2) |  | 1,604 (1,571, 1,639) | | 48.1 (47.1, 49.2) | 0.7 (-0.8, 2.3) |
| 7-3-2 | 172 (165, 180) | -79 (-87, -71) | 198 (178, 217) | 178 (160, 195) | | 5.6 (5.0, 6.1) |  | 1,578 (1,545, 1,611) | | 47.3 (46.3, 48.3) | -1.1 (-2.7, 0.4) |
| 8-3-2 | 191 (183, 199) | -61 (-69, -52) | 152 (131, 172) | 137 (118, 155) | | 4.3 (3.7, 4.9) |  | 1,529 (1,498, 1,561) | | 45.9 (44.9, 46.8) | -3.9 (-5.4, -2.4) |
| 6-4-1 | 81 (77, 84) | -171 (-174, -167) | 427 (418, 436) | 384 (376, 392) | | 12.1 (11.8, 12.3) |  | 2,072 (2,023, 2,123) | | 62.2 (60.7, 63.7) | 20.2 (18.4, 21.9) |
| 7-4-1 | 95 (91, 99) | -157 (-160, -152) | 392 (381, 402) | 353 (343, 361) | | 11.1 (10.8, 11.4) |  | 2,020 (1,973, 2,068) | | 60.6 (59.2, 62.0) | 17.6 (15.9, 19.3) |
| 8-4-1 | 110 (106, 115) | -141 (-145, -137) | 353 (342, 364) | 318 (307, 327) | | 10.0 (9.7, 10.3) |  | 1,988 (1,942, 2,035) | | 59.6 (58.3, 61.0) | 15.6 (13.9, 17.3) |
| 6-4-2 | 177 (169, 185) | -75 (-82, -67) | 187 (167, 206) | 168 (150, 185) | | 5.3 (4.7, 5.8) |  | 1,520 (1,489, 1,552) | | 45.6 (44.7, 46.5) | -3.2 (-4.7, -1.7) |
| 7-4-2 | 192 (184, 201) | -59 (-67, -50) | 148 (126, 168) | 133 (113, 151) | | 4.2 (3.6, 4.7) |  | 1,458 (1,429, 1,487) | | 43.7 (42.9, 44.6) | -6.2 (-7.6, -4.7) |
| 8-4-2 | 203 (195, 212) | -48 (-56, -39) | 120 (98, 140) | 108 (89, 126) | | 3.4 (2.8, 4.0) |  | 1,452 (1,423, 1,481) | | 43.6 (42.7, 44.4) | -7.1 (-8.6, -5.7) |
| 6-5-1 | 119 (114, 124) | -132 (-137, -127) | 332 (318, 343) | 298 (286, 309) | | 9.4 (9.0, 9.7) |  | 1,940 (1,897, 1,985) | | 58.2 (56.9, 59.5) | 13.5 (11.8, 15.2) |
| 7-5-1 | 122 (117, 127) | -130 (-134, -124) | 324 (311, 336) | 292 (280, 302) | | 9.2 (8.8, 9.5) |  | 1,923 (1,881, 1,967) | | 57.7 (56.4, 59.0) | 12.8 (11.1, 14.4) |
| 8-5-1 | 137 (132, 143) | -114 (-119, -109) | 286 (272, 298) | 257 (245, 268) | | 8.1 (7.7, 8.4) |  | 1,891 (1,850, 1,934) | | 56.7 (55.5, 58.0) | 10.7 (9.1, 12.4) |
| 6-5-2 | 212 (203, 221) | -40 (-48, -30) | 99 (75, 121) | 89 (68, 109) | | 2.8 (2.1, 3.4) |  | 1,405 (1,379, 1,432) | | 42.1 (41.4, 42.9) | -9.1 (-10.6, -7.7) |
| 7-5-2 | 216 (208, 226) | -35 (-44, -26) | 88 (64, 109) | 79 (58, 98) | | 2.5 (1.8, 3.1) |  | 1,378 (1,354, 1,404) | | 41.4 (40.6, 42.1) | -10.2 (-11.6, -8.9) |
| 8-5-2 | 227 (219, 237) | -24 (-33, -15) | 60 (37, 82) | 54 (33, 74) | | 1.7 (1.0, 2.3) |  | 1,372 (1,347, 1,398) | | 41.2 (40.4, 41.9) | -11.2 (-12.6, -9.8) |
| Risk cut-offs: 2.5% 7.5% | |  |  |  |  | |  |  |  | |  |
| 6-3-1 | 89 (85, 95) | -162 (-166, -156) | 407 (392, 417) | 366 (352, 375) | | 11.5 (11.1, 11.8) |  | 1,697 (1,653, 1,740) | | 50.9 (49.6, 52.2) | 8.3 (6.6, 9.9) |
| 7-3-1 | 104 (100, 110) | -148 (-152, -141) | 369 (354, 380) | 332 (318, 342) | | 10.4 (10.0, 10.7) |  | 1,688 (1,645, 1,731) | | 50.6 (49.3, 51.9) | 7.0 (5.3, 8.6) |
| 8-3-1 | 129 (124, 136) | -122 (-127, -115) | 306 (288, 318) | 275 (259, 286) | | 8.6 (8.1, 9.0) |  | 1,603 (1,563, 1,644) | | 48.1 (46.9, 49.3) | 2.7 (1.0, 4.2) |
| 6-3-2 | 170 (161, 181) | -81 (-90, -70) | 203 (176, 226) | 183 (158, 203) | | 5.7 (5.0, 6.4) |  | 1,488 (1,458, 1,516) | | 44.6 (43.8, 45.5) | -3.7 (-5.3, -2.2) |
| 7-3-2 | 186 (177, 197) | -65 (-74, -54) | 163 (135, 186) | 147 (121, 168) | | 4.6 (3.8, 5.3) |  | 1,476 (1,447, 1,504) | | 44.3 (43.4, 45.1) | -5.2 (-6.8, -3.7) |
| 8-3-2 | 209 (199, 221) | -42 (-52, -30) | 106 (76, 130) | 95 (69, 117) | | 3.0 (2.2, 3.7) |  | 1,399 (1,372, 1,425) | | 42.0 (41.2, 42.8) | -9.1 (-10.8, -7.6) |
| 6-4-1 | 139 (133, 147) | -113 (-118, -105) | 282 (262, 296) | 253 (235, 266) | | 8.0 (7.4, 8.4) |  | 1,512 (1,476, 1,549) | | 45.4 (44.3, 46.5) | -0.7 (-2.4, 0.8) |
| 7-4-1 | 155 (149, 164) | -96 (-102, -88) | 241 (219, 256) | 216 (197, 230) | | 6.8 (6.2, 7.2) |  | 1,442 (1,409, 1,477) | | 43.3 (42.3, 44.3) | -4.0 (-5.6, -2.5) |
| 8-4-1 | 169 (163, 178) | -82 (-88, -73) | 205 (184, 221) | 185 (165, 199) | | 5.8 (5.2, 6.2) |  | 1,426 (1,393, 1,460) | | 42.8 (41.8, 43.8) | -5.5 (-7.1, -4.0) |
| 6-4-2 | 210 (200, 222) | -42 (-51, -29) | 104 (74, 129) | 94 (66, 116) | | 2.9 (2.1, 3.6) |  | 1,330 (1,305, 1,355) | | 39.9 (39.2, 40.6) | -11.2 (-12.8, -9.8) |
| 7-4-2 | 227 (217, 240) | -24 (-34, -11) | 60 (28, 86) | 54 (26, 77) | | 1.7 (0.8, 2.4) |  | 1,258 (1,236, 1,280) | | 37.7 (37.1, 38.4) | -14.6 (-16.2, -13.2) |
| 8-4-2 | 239 (229, 251) | -13 (-23, 0) | 31 (0, 57) | 28 (0, 51) | | 0.9 (0.0, 1.6) |  | 1,249 (1,226, 1,271) | | 37.5 (36.8, 38.1) | -15.7 (-17.3, -14.3) |
| 6-5-1 | 208 (200, 219) | -43 (-51, -32) | 108 (80, 128) | 97 (72, 115) | | 3.0 (2.3, 3.6) |  | 1,354 (1,323, 1,385) | | 40.6 (39.7, 41.6) | -10.4 (-12.1, -8.9) |
| 7-5-1 | 208 (201, 219) | -43 (-51, -32) | 107 (81, 127) | 96 (73, 114) | | 3.0 (2.3, 3.6) |  | 1,343 (1,312, 1,374) | | 40.3 (39.4, 41.2) | -10.7 (-12.4, -9.3) |
| 8-5-1 | 223 (215, 233) | -29 (-37, -18) | 72 (45, 91) | 65 (41, 82) | | 2.0 (1.3, 2.6) |  | 1,327 (1,296, 1,357) | | 39.8 (38.9, 40.7) | -12.2 (-13.9, -10.8) |
| 6-5-2 | 271 (260, 286) | 20 (8, 35) | -50 (-86, -21) | -45 (-78, -19) | | -1.4 (-2.4, -0.6) |  | 1,191 (1,170, 1,211) | | 35.7 (35.1, 36.3) | -19.8 (-21.4, -18.3) |
| 7-5-2 | 273 (261, 287) | 21 (10, 35) | -54 (-89, -25) | -48 (-80, -22) | | -1.5 (-2.5, -0.7) |  | 1,177 (1,157, 1,196) | | 35.3 (34.7, 35.9) | -20.3 (-21.9, -18.9) |
| 8-5-2 | 284 (273, 298) | 33 (22, 47) | -82 (-117, -54) | -74 (-105, -48) | | -2.3 (-3.3, -1.5) |  | 1,168 (1,147, 1,187) | | 35.0 (34.4, 35.6) | -21.4 (-23.0, -20.0) |
| Risk cut-offs: 5.0% 7.5% | |  |  |  |  | |  |  |  | |  |
| 6-3-1 | 134 (128, 141) | -117 (-123, -110) | 294 (276, 308) | 264 (248, 277) | | 8.3 (7.8, 8.7) |  | 1,388 (1,349, 1,430) | | 41.6 (40.5, 42.9) | -4.1 (-5.8, -2.4) |
| 7-3-1 | 166 (160, 174) | -85 (-91, -77) | 213 (193, 229) | 192 (174, 206) | | 6.0 (5.5, 6.5) |  | 1,362 (1,324, 1,403) | | 40.9 (39.7, 42.1) | -7.2 (-8.9, -5.5) |
| 8-3-1 | 220 (213, 230) | -31 (-38, -21) | 77 (54, 96) | 70 (48, 87) | | 2.2 (1.5, 2.7) |  | 1,297 (1,262, 1,334) | | 38.9 (37.9, 40.0) | -13.0 (-14.7, -11.3) |
| 6-3-2 | 206 (196, 218) | -45 (-55, -33) | 113 (83, 138) | 101 (75, 124) | | 3.2 (2.4, 3.9) |  | 1,210 (1,185, 1,235) | | 36.3 (35.6, 37.1) | -14.6 (-16.2, -13.1) |
| 7-3-2 | 240 (229, 252) | -11 (-22, 1) | 29 (-2, 56) | 26 (-2, 50) | | 0.8 (-0.1, 1.6) |  | 1,181 (1,157, 1,205) | | 35.4 (34.7, 36.1) | -17.8 (-19.4, -16.3) |
| 8-3-2 | 288 (277, 302) | 37 (25, 50) | -93 (-126, -63) | -83 (-114, -57) | | -2.6 (-3.6, -1.8) |  | 1,131 (1,109, 1,153) | | 33.9 (33.3, 34.6) | -22.8 (-24.4, -21.3) |
| 6-4-1 | 175 (168, 184) | -76 (-83, -67) | 190 (168, 208) | 171 (151, 187) | | 5.4 (4.7, 5.9) |  | 1,329 (1,294, 1,366) | | 39.9 (38.8, 41.0) | -8.8 (-10.5, -7.2) |
| 7-4-1 | 209 (201, 218) | -43 (-51, -33) | 106 (82, 127) | 96 (74, 114) | | 3.0 (2.3, 3.6) |  | 1,278 (1,246, 1,313) | | 38.4 (37.4, 39.4) | -12.7 (-14.4, -11.1) |
| 8-4-1 | 245 (237, 256) | -6 (-14, 4) | 15 (-11, 36) | 13 (-10, 32) | | 0.4 (-0.3, 1.0) |  | 1,244 (1,213, 1,277) | | 37.3 (36.4, 38.3) | -16.3 (-18.0, -14.7) |
| 6-4-2 | 242 (231, 255) | -9 (-20, 4) | 22 (-10, 50) | 20 (-9, 45) | | 0.6 (-0.3, 1.4) |  | 1,164 (1,142, 1,187) | | 34.9 (34.3, 35.6) | -18.5 (-20.1, -17.0) |
| 7-4-2 | 277 (265, 291) | 26 (14, 40) | -65 (-99, -34) | -58 (-89, -31) | | -1.8 (-2.8, -1.0) |  | 1,110 (1,091, 1,130) | | 33.3 (32.7, 33.9) | -22.6 (-24.1, -21.1) |
| 8-4-2 | 308 (296, 322) | 57 (45, 70) | -142 (-176, -112) | -128 (-159, -101) | | -4.0 (-5.0, -3.2) |  | 1,092 (1,072, 1,111) | | 32.7 (32.2, 33.3) | -25.3 (-26.9, -23.9) |
| 6-5-1 | 232 (223, 243) | -19 (-28, -8) | 48 (20, 71) | 43 (18, 64) | | 1.3 (0.6, 2.0) |  | 1,266 (1,235, 1,300) | | 38.0 (37.1, 39.0) | -14.7 (-16.4, -13.1) |
| 7-5-1 | 241 (232, 252) | -10 (-19, 0) | 26 (-1, 48) | 23 (-1, 43) | | 0.7 (0.0, 1.4) |  | 1,241 (1,211, 1,273) | | 37.2 (36.3, 38.2) | -16.1 (-17.8, -14.5) |
| 8-5-1 | 278 (268, 289) | 26 (17, 38) | -66 (-94, -43) | -60 (-85, -39) | | -1.9 (-2.7, -1.2) |  | 1,207 (1,178, 1,238) | | 36.2 (35.3, 37.1) | -19.7 (-21.4, -18.2) |
| 6-5-2 | 296 (282, 310) | 44 (31, 59) | -111 (-148, -78) | -100 (-133, -70) | | -3.1 (-4.2, -2.2) |  | 1,110 (1,092, 1,129) | | 33.3 (32.7, 33.9) | -23.9 (-25.5, -22.4) |
| 7-5-2 | 306 (293, 320) | 54 (42, 69) | -136 (-172, -104) | -123 (-155, -94) | | -3.9 (-4.9, -2.9) |  | 1,082 (1,064, 1,100) | | 32.4 (31.9, 33.0) | -25.5 (-27.0, -24.0) |
| 8-5-2 | 337 (324, 351) | 85 (73, 100) | -214 (-250, -182) | -192 (-225, -164) | | -6.0 (-7.1, -5.1) |  | 1,063 (1,045, 1,081) | | 31.9 (31.3, 32.4) | -28.2 (-29.8, -26.8) |

QALY: quality-adjusted life year; CN¥: Chinese Yuan.

Data are estimated with 95% CIs over a 10-year period. The 10-year risks were estimated by CKB-ASCVD hard outcome model.
